# Mapping Publication Trends and Identifying Hot Spots of Research on Internet Health Information Seeking Behavior: A Quantitative and Co-Word Biclustering Analysis

**DOI:** 10.2196/jmir.3326

**Published:** 2015-03-25

**Authors:** Fan Li, Min Li, Peng Guan, Shuang Ma, Lei Cui

**Affiliations:** ^1^Department of Medical InformaticsChina Medical UniversityShenyangChina; ^2^Heping HospitalQiqiharChina; ^3^Department of EpidemiologySchool of Public HealthChina Medical UniversityShenyangChina; ^4^Department of NeurologyThe Affiliated Shengjing Hospital of China Medical UniversityShenyangChina

**Keywords:** information seeking behavior, Internet, health information, bibliometric analysis, co-word analysis, biclustering, hot spots, publication status

## Abstract

**Background:**

The Internet has become an established source of health information for people seeking health information. In recent years, research on the health information seeking behavior of Internet users has become an increasingly important scholarly focus. However, there have been no long-term bibliometric studies to date on Internet health information seeking behavior.

**Objective:**

The purpose of this study was to map publication trends and explore research hot spots of Internet health information seeking behavior.

**Methods:**

A bibliometric analysis based on PubMed was conducted to investigate the publication trends of research on Internet health information seeking behavior. For the included publications, the annual publication number, the distribution of countries, authors, languages, journals, and annual distribution of highly frequent major MeSH (Medical Subject Headings) terms were determined. Furthermore, co-word biclustering analysis of highly frequent major MeSH terms was utilized to detect the hot spots in this field.

**Results:**

A total of 533 publications were included. The research output was gradually increasing. There were five authors who published four or more articles individually. A total of 271 included publications (50.8%) were written by authors from the United States, and 516 of the 533 articles (96.8%) were published in English. The eight most active journals published 34.1% (182/533) of the publications on this topic. Ten research hot spots were found: (1) behavior of Internet health information seeking about HIV infection or sexually transmitted diseases, (2) Internet health information seeking behavior of students, (3) behavior of Internet health information seeking via mobile phone and its apps, (4) physicians’ utilization of Internet medical resources, (5) utilization of social media by parents, (6) Internet health information seeking behavior of patients with cancer (mainly breast cancer), (7) trust in or satisfaction with Web-based health information by consumers, (8) interaction between Internet utilization and physician-patient communication or relationship, (9) preference and computer literacy of people using search engines or other Web-based systems, and (10) attitude of people (especially adolescents) when seeking health information via the Internet.

**Conclusions:**

The 10 major research hot spots could provide some hints for researchers when launching new projects. The output of research on Internet health information seeking behavior is gradually increasing. Compared to the United States, the relatively small number of publications indexed by PubMed from other developed and developing countries indicates to some extent that the field might be still underdeveloped in many countries. More studies on Internet health information seeking behavior could give some references for health information providers.

## Introduction

In recent decades, Internet technology has developed rapidly and has become an important part of the daily lives of many people around the world. Meanwhile, interest in the medium as a communication tool for health-related information is growing rapidly [1-4].

The Internet has huge potential to meet the health information needs and enhance the health literacy of people because of its abundant resources, convenient access, low cost, interactivity, continuing evolution, and so on. The proliferation of the medium has, it can be argued, changed the way that people use information to protect their health [5]. To launch global health promotion campaigns and meet the increasingly urgent health information needs of everyone, more and more institutions including governments, academic organizations, medical and educational departments, and business corporations have established health information portals, with the result that Web-based health information gets richer and richer. Accordingly, people increasingly seek health information via the Internet, the fastest growing carrier of health information and the largest medical library in the world. As early as 2004, it was determined that about 4.5% of Internet searches in the world were related to health [6]. As the Internet develops, however, there is far more data than before. The latest (2013) national survey by the Pew Internet & American Life Project shows that clinicians are still the top source of health information in the United States, but Web-based information, curated by peers, is a significant supplement. Among US adults, 81% use the Internet, 59% say they have looked online for health information in the past year, and 35% say they have gone online specifically to try to figure out what medical condition they or someone else might have [7]. The statistical results indicated that more and more Internet users, not satisfied with health information provided by medical professionals, search for health information on the Internet and also enjoy the efficiency and convenience of the Internet.

In academia, health information seeking behavior is defined by scholars as “the behavior that the public or consumers exhibit when acquiring health information” [8]. As people increasingly use the Internet to seek health information in recent years, theoretical and empirical studies on Internet health information seeking behavior have been conducted by researchers. Findings of such studies are valuable for practice, such as (1) construction of all types of health or medical information websites, (2) optimization of search language of Web-based health information, (3) development of tools enhancing the users’ ability to read professional health content, (4) formulation of health information dissemination strategy aimed at specific populations, and (5) improvement of the efficiency and quality of Web-based health information services [9].

Despite the fact that scholars have published papers on Internet health information seeking behavior worldwide, there have been only several reviews about the research advancements in this field. In a short review of literature by Younger, for example, some of the existing evidence was collected from 1995 to 2009 to establish whether there were any significant differences in the ways and reasons why doctors and nurses sought out Web-based information and to establish how nurses and doctors located information online [10]. A literature review by the European Centre for Disease Prevention and Control concentrated on research articles published in English on Internet health information seeking behavior by adults from 2006 to 2010. It mainly documented Internet accessibility and usage patterns, outlined Internet health information consumer profiles, identified Internet sources of health information, outlined health professionals’ Internet use, and ascertained challenges for health professionals posed by Internet use [11].

Although the above reviews can reflect the research status on Internet health information seeking behavior to some extent, the contents and the points of view are quite different among different scholars and the bibliometric research across a long-term span is lacking. The bibliometric method, a type of quantitative analysis, has been widely used for the determination of scientific research evolution in recent years. The statistical indicators that measure the contribution of scientific publications within a given topic or research field can represent the research trends and hot spots [12]. A research hot spot refers to a focus of research for which researchers have carried out many studies and published many related papers. By computing the frequencies and relationship of words reflecting the content of articles that appear in a field, the hot spots of the field can usually be identified [13,14]. Co-word analysis is a type of bibliometric method to identify hot spots and find knowledge in academic literature, proposed as early as the late 1970s by French bibliometric scientists [15]. Its principle is as follows: if two professional terms expressing a particular research subject appear in the same articles simultaneously, these two terms may have a certain intrinsic relationship. The more frequently these two terms occur in the same articles, the closer their relationship is. According to this ‘‘distance’’, the important keywords of a subject are classified further to sum up the research focus and structure of a discipline by modern statistical techniques, such as cluster analysis, factor analysis, multidimensional scaling analysis, or multivariate analysis. It is well known that cluster analysis has been widely used to extract research themes of a field. For example, with clustering algorithms, Raghupathi et al identified the sub-fields of research of health information systems [16], and Schuemie et al characterized the domain of medical informatics [17]. Biclustering, unlike traditional clustering, allows simultaneous clustering of the rows and columns of a matrix, not only to cluster the global information, but also to find local information efficiently in high dimensional data [18]. In 1972, Hartigan first proposed the idea that simultaneous clustering could be performed for the rows and columns of a matrix [19]. Until 2000, Cheng and Church formally presented the algorithm and model of biclustering [18]. Since then, more and more excellent algorithms and models of biclustering have been developed. In recent years, biclustering analysis was introduced to the bibliometric field. Cui et al applied biclustering to analyze Chinese education status on medical informatics from the two aspects of institutes and themes [20]. Yu et al revealed the research subject areas and hot spots in biomedical informatics by biclustering analysis [21]. Fang et al performed biclustering to explore high producing authors and research features of library science and informatics in China [22]. Their research results showed that major research hot spots and representative publications or studies in one subject area could be captured with the biclustering method.

To the authors’ knowledge, at present, there have been few bibliometric articles on Internet health information seeking behavior. In the present study, a comprehensive analysis on outer characteristics and content patterns of relevant publications was carried out to reveal the research history and status in this field. Specifically, co-word biclustering analysis was utilized to identify the research hot spots of Internet health information seeking behavior. We hope this paper can provide some reference for future research on Internet health information seeking behavior.

## Methods

### Data Collection

Relevant articles were identified by searching PubMed without the restriction of language or publication year. PubMed was selected as the data source for two reasons: (1) PubMed is a free authoritative medical literature database of the National Library of Medicine on the Web, with articles on health and medical information seeking behaviors, and (2) articles from Medline (a subset of PubMed) are indexed with MeSH (Medical Subject Headings) terms, a set of normalized words that can reflect the contents of articles; based on those words, the co-word clustering analysis can be performed. The search was carried out on September 30, 2014 to ensure the search results were as current as possible. The search strategy is detailed as follows (for details on how PubMed translates this search strategy, see Multimedia Appendix 1): #1 Internet information seeking behavior; #2 Internet information seeking behaviour; #3 ((search*[ti] OR seek*[ti]) OR (behaviour[ti] OR behavior[ti])) AND (Internet[ti] OR net*[ti] OR online[ti] OR web*[ti]); #4 #1 OR #2 OR #3.

Based on the above search strategy, 2741 publications were found in PubMed. The titles and abstracts of the publications were screened according to relevance and selection criteria. The inclusion criteria were (1) the contents of papers primarily focus on Internet health information seeking behavior, and (2) all study designs. The exclusion criteria were (1) studies focus on accuracy or validity of information resources rather than Internet health information seeking behavior (eg, the article entitled “Infant teething information on the World Wide Web: taking a byte out of the search” has been excluded, because the purpose of this study was to describe and evaluate the quality of infant teething information on selected popular parenting websites [23]), (2) studies on other behaviors, such as suicidal behavior [24], Internet-based problem shopping behavior [25], Internet addictive behavior [26], regular gaming behavior [27], and so on, (3) studies on seeking other information unrelated to health, and (4) studies on other networks such as Bayesian networks [28] and metabolic networks [29] rather than the Internet. Two researchers independently reviewed and evaluated the studies and reached consensus on the inclusion for analysis. The concordance rate between them was 0.90, indicating a strong agreement [30]. Any discrepancies were discussed with reference to the research objective until consensus was reached. Initially, 494 related articles were identified. Then, we counted the number of related articles in each journal. It was found that the top three journals were, in order, the Journal of Medical Internet Research, the Journal of Health Communication, and the Journal of the Medical Library Association: JMLA, for which manual searches were also conducted and another 39 related papers were identified. Finally, a total of 533 papers were included in this study. Each publication downloaded from PubMed contained the following key eligibility items: title, author, institution, country, source, publication year, and MeSH terms. These data were saved as two files in XML and MEDLINE formats, respectively.

### Data Extraction and Analysis

Bibliographic Item Co-Occurrence Matrix Builder (BICOMB) (developed by Professor Cui from China Medical University and available freely online) [31], GoPubMed [32], and Microsoft Excel were employed to determine the distribution of the publication year, countries, authors, languages, journals, and the frequency ranking of major MeSH terms of the included publications. In this study, the most active journals were identified according to Bradford’s Law (Bradford’s Law of Scattering was first described by Bradford in 1934). The law indicates that journals in a given subject area can be sorted by the number of articles into three parts, each with about one-third of all articles: (1) a core of a few journals, (2) a second zone, with more journals, and (3) a third zone, with the bulk of journals. The number of journals in each group will be proportional to 1: n: n^2^. Although Bradford’s Law is not statistically accurate, librarians commonly use it as a guideline in the research of core journals [33].

Considering the papers included in this study had to be related to Internet health information seeking behavior, the two highly frequent MeSH terms, “Internet” and “Information Seeking Behavior”, were deleted for they would be meaningless in the content analysis. The trends of the remaining highly frequent major MeSH terms over time were illustrated visually. In order to explore the hot spots of Internet health information seeking behavior, biclustering for highly frequent major MeSH terms and included papers was performed. By biclustering, in this study, the relationship among highly frequent words and the relationship between highly frequent words and source articles could be gained. Subsequently, a binary matrix with highly frequent major MeSH terms as the rows and source articles as the columns was built from BICOMB for further biclustering by using the software “gCLUTO”, version 1.0 (Graphical CLUstering TOolkit, a graphical front-end for the CLUTO data clustering library, developed by Rasmussen, Newman, and Karypis from University of Minnesota) [34]. Parameters of biclustering in gCLUTO were set according to those appropriate for biclustering analysis based on literature [35]. Repeated bisection was chosen for clustering method, cosine for similarity function, and *I*2 for criterion function of clustering. In the hopes of being able to distinguish the optimal number of clusters, we reran the biclustering with different numbers of clusters. The biclustering result of the matrix of highly frequent major MeSH terms-source articles was shown through mountain visualization and matrix visualization. With the help of semantic relationships among MeSH terms and the content of the representative papers in each cluster, the basic framework of research hot spots of Internet health information seeking behavior was drawn and analyzed.

## Results

### Growth of the Literature

**Figure 1:**
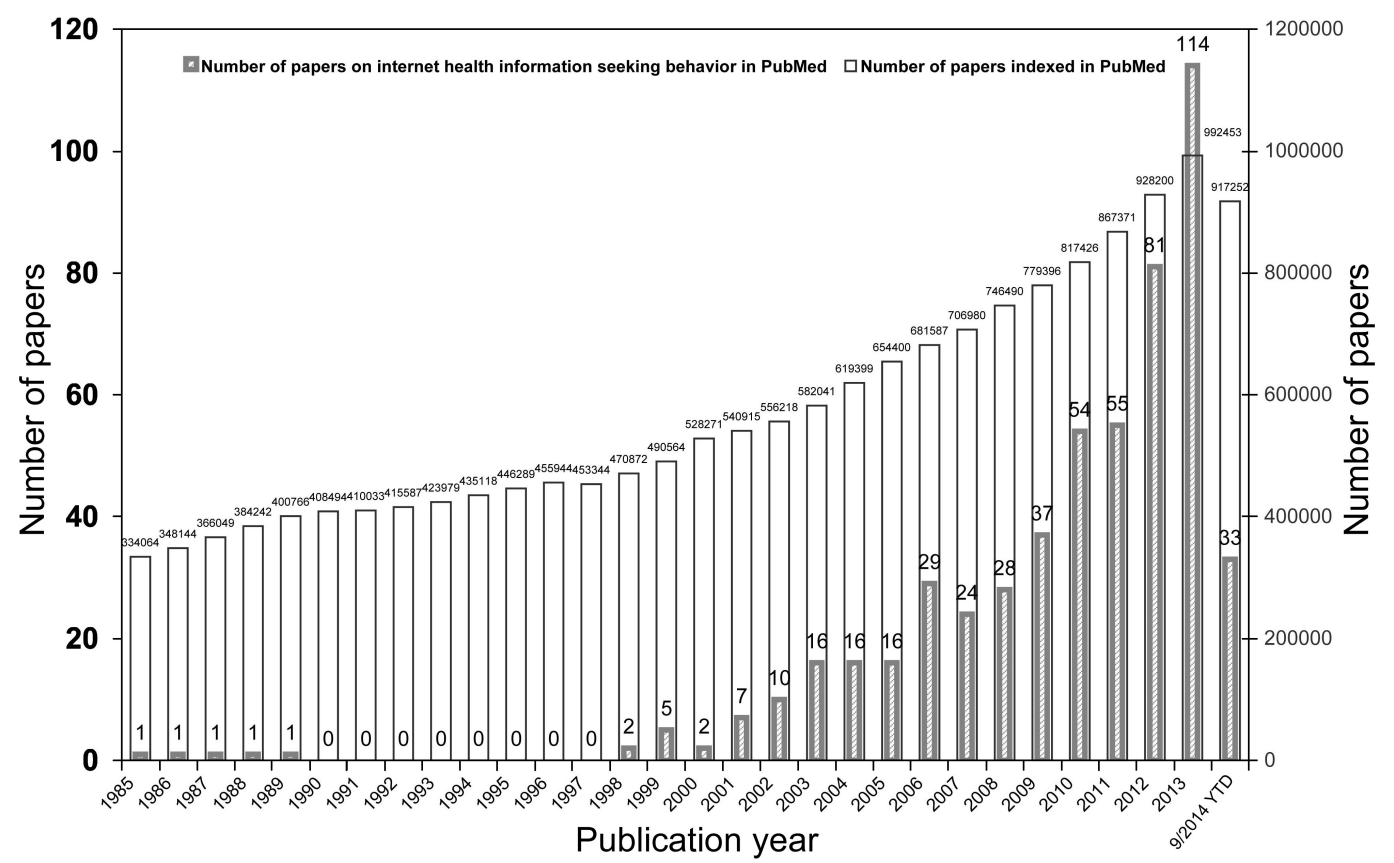
Temporal distribution of papers on Internet health information seeking behavior in PubMed and all papers indexed in PubMed.

Based on the search strategy and inclusion criteria, a total of 533 publications (see Multimedia Appendix 2) were included in this study. The average number of these publications per year was about 18. The first article was published in 1985 [36]. In Figure 1, the distribution of the publication year of articles on Internet health information seeking behavior is shown, and, in parallel, that of all the publications indexed in PubMed is also illustrated for the aim of comparison.

### Distribution of Countries, Authors, and Languages

**Figure 2:**
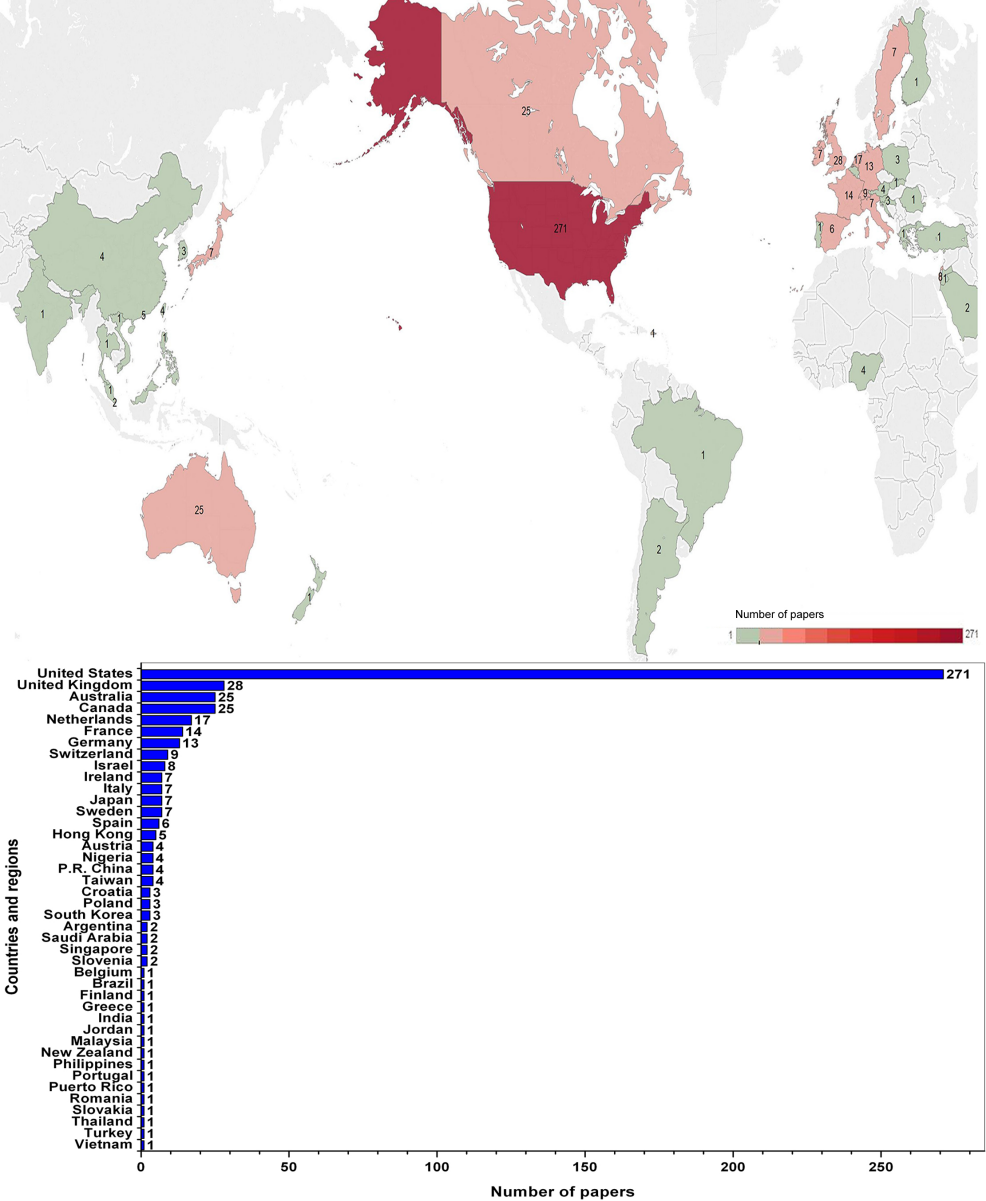
Geographic distribution of research output on Internet health information seeking behavior.

Of all articles on Internet health information seeking behavior from PubMed, 93.4% (498/533) provided the addresses of authors. So, according to the rough statistics, articles on Internet health information seeking behavior indexed in PubMed originated from at least 42 different countries or regions. Figure 2 illustrates the number of research outputs on Internet health information seeking behavior in different countries. The number in the map is the amount of related publications for every country or territory. The United States ranked first with 271 publications (50.8%).

Among all 1758 authors involved in this topic, five authors published four or more articles individually. Park ranked first for publishing five articles, of which he published one article as the first author. Dickerson published four articles for second place, yet he had three articles as the first author. The authors of the first publication on Internet health information seeking behavior were Tolle and Hah from the United States.

Most of these articles were published in English (96.8%, 516/533), the remaining articles were published in Spanish (1.1%, 6/533), German (0.8%, 4/533), French (0.6%, 3/533), Polish (0.4%, 2/533), Hebrew (0.2%, 1/533), Swedish (0.2%, 1/533), and Japanese (0.2%, 1/533), respectively. (Note, one article was published in both English and French [37].)

### Most Active Journals

Altogether, 253 journals have been involved in the field. From 1985 to 2014, the eight most active journals published 182 publications on Internet health information seeking behavior, accounting for 34.1% of all 533 publications. Table 1 displays the eight most productive journals, which are considered as the core journals in the research area of Internet health information seeking behavior under Bradford’s Law.

**Table 1:** Most active journals on the topic of Internet health information seeking behavior (PubMed sourced until September 2014) (n=533).

No.	Top journals	Publications n (%)
1	Journal of Medical Internet Research	67 (12.6)
2	Journal of Health Communication	41 (7.7)
3	Journal of the Medical Library Association: JMLA	16 (3.0)
4	American Medical Informatics Association (AMIA): Annual Symposium proceedings / AMIA Symposium. AMIA Symposium	14 (2.6)
5	CyberPsychology & Behavior: the impact of the Internet, multimedia and virtual reality on behavior and society	13 (2.4)
6	International Journal of Medical Informatics	13 (2.4)
7	PLOS ONE	9 (1.7)
8	Health Communication	9 (1.7)
Total		182 (34.1)

### Research Hot Spots of Internet Health Information Seeking Behavior

**Figure 3:**
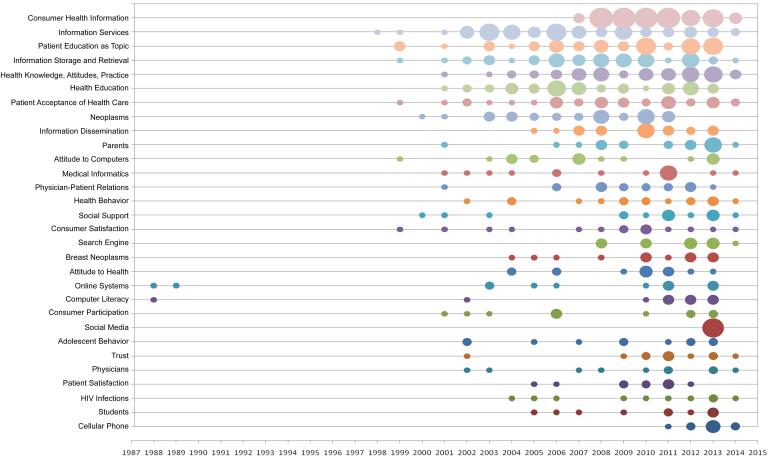
Annual distribution of highly frequent major MeSH terms from the included publications on Internet health information seeking behavior.

For publications from 1985 to September 2014, there were 495 major MeSH terms with a cumulative frequency of 1577 times excluding the two terms, “Internet” and “Information Seeking Behavior”. After discussion, a major MeSH term with a frequency of 10 or more times’ occurrence was defined as a highly frequent one. Then, 30 highly frequent major MeSH terms were extracted from the included publications with a cumulative percentage of 45.34% (715/1577) (Table 2). The annual distribution of these MeSH terms is displayed in Figure 3, where the circle indicates there is at least one publication in the corresponding year. The bigger the circle is, the more publications there are in the corresponding year. According to the situation of co-occurrence of these highly frequent MeSH terms in the same articles, a matrix was established with highly frequent major MeSH terms as the row names and source articles as the column names. The matrix (localized view in Table 3) presents the availability of the major MeSH terms in the source articles. A “1” in the cells indicates that the major MeSH term is present in the article, while “0” means absent.

**Figure 4:**
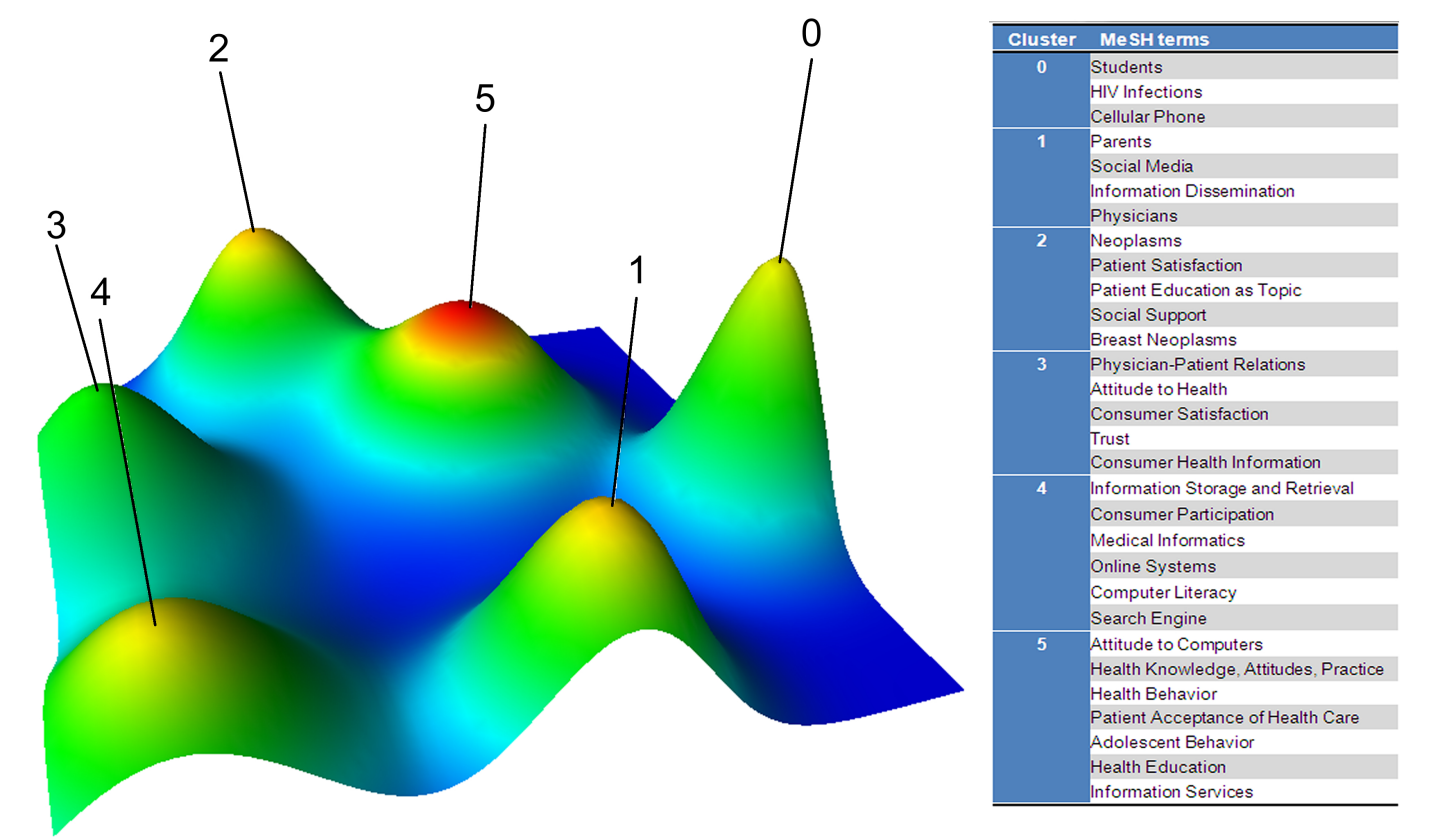
Mountain visualization of biclustering of highly frequent major MeSH terms and articles on Internet health information seeking behavior.

**Figure 5:**
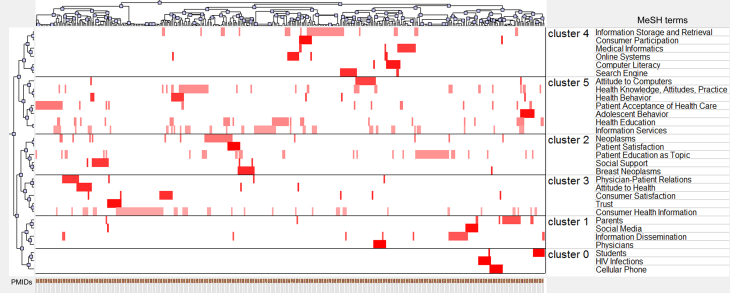
Visualized matrix of biclustering of highly frequent major MeSH terms and PubMed Unique Identifiers (PMIDs) of articles on Internet health information seeking behavior.

Biclustering was performed with the different numbers of clusters; the biclustering result of the matrix of highly frequent major MeSH terms-source articles is shown as mountain visualization and matrix visualization. Figure 4 illustrates the mountain visualization and the highly frequent MeSH terms in each cluster when these MeSH terms were divided into six clusters. The purpose of the mountain visualization is to visually aid the user in understanding the contents of a high-dimensional dataset and the efficacy effect of clustering. In Figure 4, each cluster is represented as a peak in the 3D terrain labeled by the cluster number (0 through 5, total six clusters in this study). A peak’s location on the plane, volume, height, and color are used to portray information about the associated cluster. The most informative attribute of a peak is its location on the plane with respect to other peaks. The distance between a pair of peaks on the plane represents the relative similarity of their clusters. The height of each peak is proportional to the cluster’s internal similarity. The volume of a peak is proportional to the number of MeSH terms contained within the cluster. Last, the color of a peak represents the internal standard deviation of a cluster’s objects. Red represents low deviation, whereas blue represents high deviation. According to the authors’ knowledge, each independent cluster should cover at least 30 publications and there should be no triplet peaks in the mountain visualization, thus these highly frequent MeSH terms were divided into six clusters. Figure 5 shows the matrix visualization, in which the row labels stand for highly frequent major MeSH terms, and the column labels are PubMed Unique Identifiers (PMIDs) of source articles, respectively on the right and the bottom of the matrix. Colors are used to graphically represent the values present in the matrix. The color of each grid represents the relative occurrence frequency of a MeSH term in an article. The increasingly darker shade of red represents larger values and the white color represents the values near zero. The rows of the primary matrix (Table 3) are reordered by biclustering, so that rows of the same cluster are aggregated; black horizontal lines separate these clusters. The matrix visualization reveals that 30 highly frequent major MeSH terms are clustered to six clusters. The left hierarchical tree depicts the relationships between highly frequent major MeSH terms, and the hierarchical tree on the top displays the relationships between articles. Also, it shows the corresponding articles in which each highly frequent MeSH term of each cluster occurs. A closer reading of the representative articles of each cluster contributed to the work of identifying and summarizing the themes of each cluster.

Moreover, some clusters could be divided into smaller topics based on the following criteria discussed by the research group: (1) the semantic relationship among the MeSH terms within one bigger cluster, (2) the year of MeSH terms being introduced in MeSH vocabulary, and (3) the categories in which the MeSH terms are located. The MeSH terms such as “Students”, “Parents”, and “Physicians” are located under the category of “Persons” or “Anthropology”. “Cellular Phone” and “Social Media” were the two newly introduced MeSH terms. Each of these smaller topics was thus summarized to a separate hot spot. Therefore, 10 hot topics in total were found in the field of Internet health information seeking behavior, as follows: (1) behavior of Internet health information seeking about HIV infection or sexually transmitted diseases (Cluster 0), (2) Internet health information seeking behavior of students (Cluster 0), (3) behavior of Internet health information seeking via mobile phone and its apps (Cluster 0), (4) physicians’ utilization of Internet medical resources (Cluster 1), (5) utilization of social media by parents (Cluster 1), (6) Internet health information seeking behavior of patients with cancer (mainly breast cancer) (Cluster 2), (7) trust in or satisfaction with Web-based health information by consumers (Cluster 3), (8) interaction between Internet utilization and physician-patient communication or relationship (Cluster 3), (9) preference and computer literacy of people using search engines or other Web-based systems (Cluster 4), and (10) attitude of people (especially adolescents) when seeking health information via the Internet (Cluster 5).

**Table 2:** Highly frequent major MeSH^a^ terms from the included publications on Internet health information seeking behavior (n=1577).

No.	MeSH terms	Frequency n (%^b^)	Cumulative percentage, %
1	Consumer Health Information	77 (4.88)	4.88
2	Information Services	67 (4.25)	9.13
3	Patient Education as Topic	55 (3.49)	12.62
4	Information Storage and Retrieval	50 (3.17)	15.79
5	Health Knowledge, Attitudes, Practice	50 (3.17)	18.96
6	Health Education	44 (2.79)	21.75
7	Patient Acceptance of Health Care	35 (2.22)	23.97
8	Neoplasms	33 (2.09)	26.06
9	Information Dissemination	23 (1.46)	27.52
10	Parents	21 (1.33)	28.85
11	Attitude to Computers	18 (1.14)	29.99
12	Medical Informatics	17 (1.08)	31.07
13	Physician-Patient Relations	16 (1.01)	32.09
14	Health Behavior	16 (1.01)	33.10
15	Social Support	16 (1.01)	34.12
16	Consumer Satisfaction	15 (0.95)	35.07
17	Search Engine	15 (0.95)	36.02
18	Breast Neoplasms	14 (0.89)	36.91
19	Attitude to Health	14 (0.89)	37.79
20	Online Systems	13 (0.82)	38.62
21	Computer Literacy	12 (0.76)	39.38
22	Consumer Participation	11 (0.70)	40.08
23	Social Media	11 (0.70)	40.77
24	Adolescent Behavior	11 (0.70)	41.47
25	Trust	11 (0.70)	42.17
26	Physicians	10 (0.63)	42.80
27	Patient Satisfaction	10 (0.63)	43.44
28	HIV Infections	10 (0.63)	44.07
29	Students	10 (0.63)	44.71
30	Cellular Phone	10 (0.63)	45.34

^a^MeSH: Medical Subject Headings

^b^Proportion of the frequency among 1577 times’ appearance.

**Table 3:** Highly frequent major MeSH^a^ terms-source articles matrix (localized).

No.	Major MeSH terms	PubMed Unique Identifiers of source articles				
		10052399	10402805	10590585	…	9934530
1	Consumer Health Information	0	0	0	…	0
2	Information Services	0	0	0	…	0
3	Patient Education as Topic	1	1	1	…	0
4	Information Storage and Retrieval	0	0	0	…	1
…	…	…	…	…	…	…
29	Students	0	0	0		0
30	Cellular Phone	0	0	0	…	0

^a^MeSH: Medical Subject Headings

## Discussion

### Principal Findings

In the context of the development of the Internet and its application in the field of health, academic communities have paid increasing attention to the Internet health information seeking behavior of people. With that in mind, the present study revealed an increase in publications and 10 hot spots of research on Internet health information seeking behavior from a quantitative and content point of view. Compared to the trend of all publications in PubMed, a more significant increase was found, suggesting that the research output increase of the Internet health information seeking behavior subfield was not proportional to the research output increase corresponding to the whole medical field. As expected, the number of publications was less before 2000 and started to increase during the period from 2001 to 2010s. The maximal number of publications (21.4%, 114/533) was found in 2013. In the publications included, the first one on Internet health information seeking behavior can be traced back to 1985; the authors were Americans, suggesting that investigation in this field may have originated in the United States. The authors of this article examined user search patterns on the CATLINE database [36]. Currently, the United States is still the top producer of papers in the field. In terms of all producer countries, the major contributions were from a small number of developed countries in North America, Europe, and Australia. This may be due to the fact that there is more awareness and a higher level of Internet health information seeking behavior research in those countries, where researchers have relative superiority in English as well. For the authors’ native language of Chinese, relevant articles on the target topic were also searched in the China Academic Journals Full-Text Database, China Doctoral Dissertations Full-Text Database, China Masters’ Theses Full-Text Database, and China Proceedings of Conference Full-Text Database in China National Knowledge Infrastructure (CNKI), which provides comprehensive and current Chinese information on a worldwide scale, including dissertations, proceedings, academic papers, and books from a variety of publishers, research institutions, and information sources in China. In total, 21 articles (17 journal articles, one conference article, and three graduate theses) were found up to September 2014; the first batch was published in 2008. Moreover, compared to the United States, authors from other developed or developing countries/regions (such as United Kingdom, Australia, New Zealand, and India), where English is the official language or native language, did not publish an equivalent number of related articles, so it is reasonable to infer to some extent that the field is still underdeveloped in many countries. Given the free advantage of PubMed, the results of the present study could indicate some relative preferences in the related scientific community who highly rely on PubMed for accessing professional information.

In this study, it was found that the eight most active journals had published 182 related publications (accounting for 34.1% of all related publications), and could be considered as the core journals in the research area of Internet health information seeking behavior. Among the list of the most active journals, the Journal of Medical Internet Research is the top most productive journal. Furthermore, it can be found that publications on Internet health information seeking behavior were mostly published in the journals on medical Internet research, health communication, behavior, psychology, and medical informatics, suggesting that the field crosses multiple disciplines and needs to include knowledge such as communication science, medicine, health promotion, social marketing, psychology, information technology, etc.

Methodologically, the biclustering method and visualization using gCLUTO software was employed to find hot topics on Internet health information seeking behavior in the present study. Altogether, 10 research hot spots of Internet health information seeking behavior were found, as follows:

1. Behavior of Internet health information seeking about HIV infection or sexually transmitted diseases. For instance, in Southern California, a probability sample of 195 YMSM (young men who have sex with men) using Grindr (a mobile phone app) were administered a Web-based survey to assess patterns of and motivations for Grindr use in order to inform development and tailoring of mobile phone-based HIV prevention for YMSM. The results showed that 70% of YMSM expressed a willingness to participate in a mobile phone app-based HIV prevention program. Development and testing of mobile phone apps for HIV prevention delivery has the potential to engage YMSM in HIV prevention programming, which can be tailored based on use patterns and motivations for use [38].

2. Internet health information seeking behavior of students. A cross-sectional study, for example, examined how personality traits such as sensation-seeking and impulsive decision making affected Taiwanese college students’ intentions to seek Web-based information [39].

3. Behavior of Internet health information seeking via mobile phone and its apps. A case in this point is the study that people used mobile phone apps for bariatric surgery [40].

4. Physicians’ utilization of Internet medical resources. For example, Shabi et al determined the extent, purpose, determinants, and the impact of the utilization of Internet medical databases among physicians [41].

5. Utilization of social media by parents. For example, in Gabbert et al’s study, they studied the experiences of parents of preterm infants using social networking sites and the potential of such sites for gathering information and facilitating personal exchange [42].

6. Internet health information seeking behavior of patients with cancer (mainly breast cancer). For example, a randomized controlled experiment examined which search facility for Web-based stories resulted in the satisfaction and search success of patients with breast cancer. It was found that having access to the story topics’ search facility clearly had the most positive effect on patient satisfaction and search success [43]. To better understand cancer patients’ Web-based information and support seeking behaviors, another study explored how various social and psychological characteristics predicted different levels of engagement with an online breast cancer support group: posters, lurkers, and nonusers. Results showed that the patterns of engagement with the cancer support group differed according to the patients’ characteristics [44].

7. Trust in or satisfaction with Web-based health information by consumers. For example, a study built on theoretical perspectives of trust such as personal capital-based, social capital-based, and transfer-based, examined various correlates of consumer trust in Web-based health information [45]. Another study examined the relationship among consumers’ motivation, perceived quality, satisfaction, and intention to repeat-search eHealth information [46].

8. Interaction between Internet utilization and physician-patient communication or relationship. Provider-patient communication is an important factor influencing patients’ satisfaction and health outcomes. Traditional physician-patient relationships are being challenged by the Internet health information seeking behavior of people. Hou et al drew upon the uses and gratification theory to examine how individuals’ perception of communication with health care providers was associated with their Internet use for health-related activities. The researchers found that as individuals perceived their communication with providers to be less patient-centered, they were more likely to engage in various types of Web-based health activities, such as using websites for healthy lifestyles, searching for health care providers, and seeking health information [47]. Another study has examined how Internet use for health information affected the frequency of contact with health professionals [48].

9. Preference and computer literacy of people using search engines or other Web-based systems. A study investigated the impact of preference for information on the search behavior of general consumers seeking health information, their perceptions of search tasks, and user experience with search systems. Those with a high preference were found more likely to use more general queries when searching for specific factual information and to develop more complex mental representations of health concerns of an exploratory nature and try different combinations of concepts to explore these concerns. High-preference users were also more demanding on the system. Health information search systems should be tailored to fit individuals’ information preferences [49].

10. Attitude of people (especially adolescents) when seeking health information via the Internet. For example, a paper explored UK and US adolescents’ perceptions and experiences using the Internet to find information about health and medicines [50].

From the annual distribution of highly frequent MeSH terms, it can be found that some topics such as Internet health information seeking behavior of patients with cancer are always the research hot spots. We also found the topic on behavior of Internet health information seeking via mobile phone and its apps was quite popular since 2011. That may be the result of the rapid development of mobile Internet in recent years.

### Limitations

Also, we recognize that there are several potential limitations that may encourage further research efforts. First, Figure 1 shows that there were no related articles from 1990 to 1997. While it may be objective, it may also be attributable to the fact that the included research output of our target field was only represented by the publications in a single database, PubMed, and external publications about this topic could not be analyzed. But these issues will be solved in future studies depending on the progress of text data mining techniques and more open access literature databases such as PsycINFO. We hope for the accessibility of more literature databases in the future, and then we can utilize the literature to do more studies with better quality. Second, 6.6% (35/533) of articles did not present complete affiliation data, so these articles were not assigned to a country. This might bring the bias of underestimation for some countries. Still, for identifying the country of origin, the method used was according to the corresponding author address, although often used, also not allowing recognition of transnational research. Last, although co-word biclustering is a quite useful method for identifying hot spots of one field, in this study, the results may be also affected by factors such as the accuracy of indexing documents with MeSH terms and the time when a MeSH term is introduced to the MeSH vocabulary.

### Conclusions

In this study, content analysis by co-word biclustering was employed to visualize research hot spots in the field of Internet health information seeking behavior. Ten research hot spots were found, which could provide some hints for researchers when launching new projects.

Other bibliometric measures also show the research status of this field; it was found that the research output on Internet health information seeking behavior is gradually increasing. The top most productive journal is the Journal of Medical Internet Research, which is the chief journal publishing articles on Internet health information seeking behavior. In addition, compared to the United States, the relatively small number of publications indexed by PubMed from other developed and developing countries indicates to some extent that the field might be still underdeveloped in many countries. More studies on Internet health information seeking behavior could give some references for health information providers.

## Multimedia Appendix 1

Study search strategy and corresponding search details of PubMed.

## Multimedia Appendix 2

List of included publications on Internet health information seeking behavior in this study.

## Abbreviations

BICOMB: Bibliographic Item Co-Occurrence Matrix Builder
MeSH: Medical Subject Headings
YMSM: young men who have sex with men
