# Supplementary material for: Mapping Publication Trends and Identifying Hot Spots of Research on Internet Health Information Seeking Behavior: A Quantitative and Co-Word Biclustering Analysis
Source: J Med Internet Res. 2015 Mar 25;17(3):e81. doi: 10.2196/jmir.3326 (PMC4390616; doi:10.2196/jmir.3326)
Supplement: Supplementary file 1 [file jmir_v17i3e81_app1.pdf]

The search strategy and the corresponding search details of PubMed in this study as below:

#1 Internet information seeking behavior

Search details of PubMed: ("internet"[MeSH Terms] OR "internet"[All Fields]) AND ("information seeking behavior"[MeSH Terms] OR ("information"[All Fields] AND "seeking"[All Fields] AND "behavior"[All Fields]) OR "information seeking behavior"[All Fields])

#2 Internet information seeking behaviour

Search details of PubMed: ("internet"[MeSH Terms] OR "internet"[All Fields]) AND information[All Fields] AND seeking[All Fields] AND ("behaviour"[All Fields] OR "behavior"[MeSH Terms] OR "behavior"[All Fields])

#3 ((search*[ti] OR seek*[ti]) OR (behaviour[ti] OR behavior[ti])) AND (Internet[ti] OR net*[ti] OR online[ti] OR web*[ti])

Search details of PubMed: (((search[ti] OR search's[ti] OR searchability[ti] OR searchable[ti] OR searchdogs[ti] OR searched[ti] OR searcher[ti] OR searcher's[ti] OR searchers[ti] OR searchers'[ti] OR searches[ti] OR searchgtr[ti] OR searchgui[ti] OR searchin[ti] OR searchin'[ti] OR searching[ti] OR searchings[ti] OR searchlight[ti] OR searchlights[ti] OR searchline[ti] OR searchomyces[ti] OR searchpatterns[ti] OR searchpattool[ti] OR searchpks[ti] OR searchs[ti] OR searchsmallrna[ti] OR searchwise[ti] OR searchxlinks[ti]) OR (seek[ti] OR seek'[ti] OR seek1[ti] OR seeker[ti] OR seeker's[ti] OR seeker2[ti] OR seekers[ti] OR seekers'[ti] OR seekership[ti] OR seekh[ti] OR seeking[ti] OR seeking'[ti] OR seekl[ti] OR seekling[ti] OR seekonk[ti] OR seekquence[ti] OR seeks[ti] OR seektb[ti])) OR (behaviour[ti] OR behavior[ti])) AND (internet[ti] OR (net[ti] OR net'[ti] OR net"[ti] OR net's[ti] OR net1[ti] OR net1a[ti] OR net2[ti] OR net25[ti] OR net2ch2[ti] OR net2p[ti] OR net3[ti] OR net37[ti] OR net39[ti] OR net3c[ti] OR net4[ti] OR net4oh[ti] OR neta[ti] OR neta5[ti] OR netabolism[ti] OR netabolites[ti] OR netacet[ti] OR netaffx[ti] OR netage[ti] OR netaji[ti] OR netal[ti] OR netalid[ti] OR netalide[ti] OR netalign[ti] OR netaligner[ti] OR netallic[ti] OR netamiftide[ti] OR netamines[ti] OR netaniahu[ti] OR netanya[ti] OR netanyahu[ti] OR netaprina[ti] OR netar[ti] OR netasa[ti] OR netastasizing[ti] OR netatlas[ti] OR netaudit[ti] OR netazepide[ti] OR netb[ti] OR netball[ti] OR netballers[ti]

OR netballers'[ti] OR netbiov[ti] OR netbooks[ti] OR netbox[ti] OR netcad[ti] OR netcage[ti] OR netcages[ti] OR netcare[ti] OR netcare's[ti] OR netcell[ti] OR netcglyc[ti] OR netchain[ti] OR netcitizens[ti] OR netclass[ti] OR netcloak[ti] OR netclust[ti] OR netcme[ti] OR netcmpt[ti] OR netcoaching[ti] OR netcoffee[ti] OR netcomm[ti] OR netcord[ti] OR netcssp[ti] OR netctl[ti] OR netctl1[ti] OR netctlpan[ti] OR netcutter[ti] OR netd[ti] OR netdiseasesnp[ti] OR netds[ti] OR netea[ti] OR netelast[ti] OR netelia[ti] OR neter[ti] OR neteri[ti] OR netero[ti] OR neterton's[ti] OR netevolve[ti] OR netfcm[ti] OR netflix[ti] OR netfluxes[ti] OR netgem[ti] OR netgen[ti] OR netgenerator[ti] OR netgestalt[ti] OR netgi[ti] OR netgirls[ti] OR netgrep[ti] OR neth[ti] OR neth's[ti] OR nethalide[ti] OR nethaphyl[ti] OR nethaprin[ti] OR nethaprin'[ti] OR nethaprine[ti] OR nethelands[ti] OR nether[ti] OR netheralands[ti] OR netherland[ti] OR netherland's[ti] OR netherlanders[ti] OR netherlandish[ti] OR netherlands[ti] OR netherlands'[ti] OR netherlands1[ti] OR netherlandsdagger[ti] OR netherlandsspecial[ti] OR netherllands[ti] OR netherne[ti] OR nethersole[ti] OR netherton[ti] OR netherton's[ti] OR netherworld[ti] OR nethlerlands[ti] OR nethling[ti] OR nethmap[ti] OR nethnography[ti] OR nethol[ti] OR nethoxy[ti] OR nethralaya[ti] OR nethravathi[ti] OR nethylmaleimide[ti] OR neti[ti] OR neti'[ti] OR netic[ti] OR neticonazole[ti] OR netics[ti] OR netilmicin[ti] OR netilmicine[ti] OR netilmycin[ti] OR netilyn[ti] OR netineti[ti] OR neting[ti] OR netiquette[ti] OR netivot[ti] OR netivudine[ti] OR netizen[ti] OR netjilik[ti] OR netl[ti] OR netl's[ti] OR netley[ti] OR netlike[ti] OR netlines[ti] OR netlogo[ti] OR netma[ti] OR netmakers[ti] OR netmatch[ti] OR netmeeting[ti] OR netmenu[ti] OR netmes[ti] OR netmetaxl[ti] OR netmets[ti] OR netmhc[ti] OR netmhccons[ti] OR netmhciipan[ti] OR netmhcpan[ti] OR netmhcstab[ti] OR netmode[ti] OR netmorph[ti] OR netmums[ti] OR netnep[ti] OR netnes[ti] OR netnographic[ti] OR netnotes[ti] OR neto[ti] OR neto1[ti] OR neto2[ti] OR netobimin[ti] OR netocyd[ti] OR netoglitazone[ti] OR netoglyc[ti] OR netolitzky[ti] OR netom[ti] OR netosis[ti] OR netou6sek[ti] OR netousek[ti] OR netousek's[ti] OR netpath[ti] OR netpathminer[ti] OR netpen[ti] OR netphiles[ti] OR netphosbac[ti] OR netphosyeast[ti] OR netplay[ti] OR netpoints[ti] OR netprints[ti] OR netprotect[ti] OR netra[ti] OR netradarpanam[ti] OR netralization[ti] OR netraroga[ti] OR netravathi[ti] OR netrave[ti] OR netrha[ti] OR netrin[ti]

OR netrin1[ti] OR netring[ti] OR netring2[ti] OR netrins[ti] OR netrium[ti] OR netromycin[ti] OR netronidazole[ti] OR netrophil[ti] OR netrophils[ti] OR netropica[ti] OR netropsin[ti] OR netropsin's[ti] OR netropsins[ti] OR netropsis[ti] OR netrosamines[ti] OR nets[ti] OR nets'[ti] OR netsad[ti] OR netscan[ti] OR netscape[ti] OR netsch[ti] OR netschaevo[ti] OR netschilluk[ti] OR netseed[ti] OR netskii[ti] OR netslim[ti] OR netsm[ti] OR netspeak's[ti] OR netspeed[ti] OR netsu[ti] OR netsuke[ti] OR netsukes[ti] OR nett[ti] OR netta[ti] OR nettab[ti] OR nettalk[ti] OR nettastomatidae[ti] OR nettastomatis[ti] OR nettdoktor[ti] OR netted[ti] OR nettel[ti] OR nettelbeck[ti] OR netten[ti] OR nettepi[ti] OR netter[ti] OR netter's[ti] OR nettersheim[ti] OR netterville[ti] OR nettesheim[ti] OR nettest[ti] OR nettie[ti] OR netting[ti] OR nettings[ti] OR nettion[ti] OR nettionis[ti] OR nettl[ti] OR nettle[ti] OR nettled[ti] OR nettleman[ti] OR nettlerash[ti] OR nettles[ti] OR nettleship[ti] OR nettleship's[ti] OR nettlesome[ti] OR nettleton[ti] OR nettling[ti] OR nettlu[ti] OR netto[ti] OR nettorhynchus[ti] OR nettorhynque[ti] OR nettuno[ti] OR netturnp[ti] OR netu[ti] OR netuma[ti] OR netumor[ti] OR netupitant[ti] OR netural[ti] OR neturophils[ti] OR netuschil[ti] OR netvenn[ti] OR netvibes[ti] OR netview[ti] OR netwalker[ti] OR netwass[ti] OR netwatch[ti] OR netweavers[ti] OR netweb[ti] OR netwell[ti] OR netwellness[ti] OR netwerk[ti] OR netwerks[ti] OR netwhat[ti] OR netwok[ti] OR netwoker[ti] OR network[ti] OR network'[ti] OR network's[ti] OR network2canvas[ti] OR networkability[ti] OR networkanalyst[ti] OR networkblast[ti] OR networkcentric[ti] OR networked[ti] OR networked'[ti] OR networker[ti] OR networker's[ti] OR networkers[ti] OR networkers'[ti] OR networkin[ti] OR networking[ti] OR networking'[ti] OR networklike[ti] OR networkmodel[ti] OR networkprioritizer[ti] OR networkry[ti] OR networks[ti] OR networks'[ti] OR networksin[ti] OR networktrail[ti] OR networkview[ti] OR networkviewer[ti] OR networkwide[ti] OR networm[ti] OR networth[ti] OR networx[ti] OR netwrok[ti] OR netz[ti] OR netzahualcoyone[ti] OR netzahualcoyotl[ti] OR netzelia[ti] OR netzhaut[ti] OR netzhautfunktionsstorungen[ti] OR netzwerk[ti] OR netzwerks[ti]) OR online[ti] OR (web[ti] OR web'[ti] OR web's[ti] OR web1[ti] OR web2[ti] OR web2086[ti] OR web2170[ti] OR web2ohs[ti] OR web3[ti] OR web3d[ti] OR webacgh[ti] OR webact[ti] OR weballergen[ti] OR webarray[ti] OR webarraydb[ti] OR webaugustus[ti] OR webb[ti] OR webb's[ti] OR

webbe[ti] OR webbe's[ti] OR webbed[ti] OR webber[ti] OR webber's[ti] OR webberi[ti] OR webbi[ti] OR webbiana[ti] OR webbianus[ti] OR webbii[ti] OR webbing[ti] OR webbings[ti] OR webbio[ti] OR webbiobank[ti] OR webblast[ti] OR webboards[ti] OR webbook[ti] OR webby[ti] OR webcam[ti] OR webcams[ti] OR webcare[ti] OR webcarma[ti] OR webcast[ti] OR webcasting[ti] OR webcasts[ti] OR webcat[ti] OR webcell[ti] OR webchem[ti] OR webcis[ti] OR webcite[ti] OR webcol[ti] OR webcoli[ti] OR webcor[ti] OR webcraft[ti] OR webcsd[ti] OR webct[ti] OR webd[ti] OR webdasc[ti] OR webdietaid[ti] OR webdip[ti] OR webdoctor[ti] OR webease[ti] OR webeav[ti] OR webencounter[ti] OR weber[ti] OR weber's[ti] OR weberb[ti] OR webergizes[ti] OR weberi[ti] OR weberian[ti] OR weberites[ti] OR webers[ti] OR webers'[ti] OR webexp[ti] OR webfarm[ti] OR webfeature[ti] OR webflow[ti] OR webfog[ti] OR webfolio[ti] OR webfr3d[ti] OR webgaging[ti] OR webgbrowse[ti] OR webgel[ti] OR webgestalt[ti] OR webgester[ti] OR webgimm[ti] OR webgis[ti] OR webgl[ti] OR webglore[ti] OR webgmap[ti] OR webgraph[ti] OR webgroups[ti] OR webhealth[ti] OR webic[ti] OR webification[ti] OR webifying[ti] OR webinar[ti] OR webinars[ti] OR webino[ti] OR webinterviewer[ti] OR webiz[ti] OR weblab[ti] OR webler[ti] OR webley[ti] OR weblike[ti] OR webliography[ti] OR weblog[ti] OR weblogo[ti] OR weblogs[ti] OR webm[ti] OR webmam[ti] OR webmap[ti] OR webmarketing[ti] OR webmaster[ti] OR webmastering[ti] OR webmasters[ti] OR webmd[ti] OR webmd's[ti] OR webmedqual[ti] OR webmga[ti] OR webmgr[ti] OR webmia[ti] OR webmicroscope[ti] OR webmill[ti] OR webmol[ti] OR webmotifs[ti] OR webmovil[ti] OR webmta[ti] OR webnests[ti] OR webneuro[ti] OR webnm[ti] OR webofscience[ti] OR webographies[ti] OR webometrics[ti] OR webometry[ti] OR weboncoll[ti] OR webonex[ti] OR webop[ti] OR weborg[ti] OR webosce[ti] OR webots[ti] OR webpad[ti] OR webpage[ti] OR webpages[ti] OR webparc[ti] OR webpare[ti] OR webpdbinder[ti] OR webphylip[ti] OR webpics[ti] OR webpipsa[ti] OR webpk[ti] OR webprank[ti] OR webprc[ti] OR webpredictor[ti] OR webprinses[ti] OR webproanalyst[ti] OR webprop[ti] OR webprotege[ti] OR webq[ti] OR webqtl[ti] OR webquest[ti] OR webquests[ti] OR webrasp[ti] OR webrds[ti] OR webreport[ti] OR webreview[ti] OR webril[ti] OR webrn[ti] OR webs[ti] OR websage[ti] OR websat[ti] OR webscipio[ti] OR websdale[ti] OR websem[ti] OR webserver[ti] OR

webservers[ti] OR webservice[ti] OR websidd[ti] OR webside[ti] OR websim[ti] OR website[ti] OR website's[ti] OR websites[ti] OR websites'[ti] OR websky[ti] OR websmr[ti] OR webspace[ti] OR webspaces[ti] OR webspinner[ti] OR webspinners[ti] OR webspinning[ti] OR webstation[ti] OR webster[ti] OR webster's[ti] OR websteri[ti] OR websters[ti] OR websters'[ti] OR webstore[ti] OR webstruct[ti] OR websuite[ti] OR websurfers[ti] OR websurg[ti] OR webtag[ti] OR webtalk[ti] OR webthal[ti] OR webtool[ti] OR webtools[ti] OR webtraceminer[ti] OR webucation[ti] OR webuye[ti] OR webvar[ti] OR webvision[ti] OR webwatch[ti] OR webways[ti] OR webwide[ti] OR webwise[ti] OR webwork[ti] OR webworm[ti] OR webworms[ti] OR webworn[ti]))

#4 # 1 OR #2 OR #3
