# Supplementary material for: Mapping Publication Trends and Identifying Hot Spots of Research on Internet Health Information Seeking Behavior: A Quantitative and Co-Word Biclustering Analysis
Source: J Med Internet Res. 2015 Mar 25;17(3):e81. doi: 10.2196/jmir.3326 (PMC4390616; doi:10.2196/jmir.3326)
Supplement: Supplementary file 2 [file jmir_v17i3e81_app2.pdf]

List of included publications on internet health information seeking behavior in this study

[1] Tolle J E, Hah S. Online search patterns: NLM CATLINE database[J]. J Am Soc Inf Sci. 1985, 36(2): 82-93.

[2] Sewell W, Teitelbaum S. Observations of end-user online searching behavior over eleven years[J]. J Am Soc Inf Sci. 1986, 37(4): 234-245.

[3] France C L, Kimmel S, Allegri F, et al. Integrating and evaluating online bibliographic searching with clinical experiences of third year medical students[J]. Res Med Educ. 1988, 27: 106-111.

[4] Marshall J G. Characteristics of early adopters of end-user online searching in the health professions[J]. Bull Med Libr Assoc. 1989, 77(1): 48-55.

[5] Pelzer N L, Wiese W H, Leysen J M. Library use and information-seeking behavior of veterinary medical students revisited in the electronic environment[J]. Bull Med Libr Assoc. 1998, 86(3): 346-355.

[6] Lomax E C, Lowe H J. Information needs research in the era of the digital medical library[J]. Proc AMIA Symp. 1998: 658-662.

[7] Eysenbach G, Diepgen T L. Patients looking for information on the Internet and seeking teleadvice: motivation, expectations, and misconceptions as expressed in e-mails sent to physicians[J]. Arch Dermatol. 1999, 135(2): 151-156.

[8] Jonsson P M, Ostman J, Carlsson C, et al.[Patients with diabetes search facts about their disease on the net. More information in Swedish is needed][J]. Lakartidningen. 1999, 96(24): 2970-2974.

[9] Patients seek medical advice on the Web[J]. Iowa Med. 1999, 89(6): 14.

[10] Pennbridge J, Moya R, Rodrigues L. Questionnaire survey of California consumers' use and rating of sources of health care information including the Internet[J]. West J Med. 1999, 171(5-6): 302-305.

[11] Ash J S. Factors affecting the diffusion of online end user literature searching[J]. Bull Med Libr Assoc. 1999, 87(1): 58-66.

[12] Dickerson S S, Flaig D M, Kennedy M C. Therapeutic connection: help seeking on the Internet for persons with implantable cardioverter defibrillators[J]. Heart Lung. 2000, 29(4): 248-255.

[13] Reeves P M. Coping in cyberspace: the impact of Internet use on the ability of HIV-positive individuals to deal with their illness[J]. J Health Commun. 2000, 5 Suppl: 47-59.

[14] Goodson P, Mccormick D, Evans A. Searching for sexually explicit materials on the Internet: an exploratory study of college students' behavior and attitudes[J]. Arch Sex Behav. 2001, 30(2): 101-118.

[15] Navarro F H, Wilkins S T. A new perspective on consumer health Web use: "valuegraphic" profiles of health information seekers[J]. Manag Care Q. 2001, 9(2): 35-43.

[16] Sing A, Salzman J, Sing D. Problems and risks of unsolicited e-mails in patient-physician encounters in travel medicine settings[J]. J Travel Med. 2001, 8(3): 109-112.

[17] Mantovani G. The psychological construction of the Internet: from information foraging to social gathering to cultural mediation[J]. Cyberpsychol Behav. 2001, 4(1): 47-56.

[18] Lathey J W, Hodge B. Information seeking behavior of occupational health nurses. How nurses keep current with health information[J]. AAOHN J. 2001, 49(2): 87-95.

[19] Cline R J, Haynes K M. Consumer health information seeking on the Internet: the state of the art[J]. Health Educ Res. 2001, 16(6): 671-692.
[20] Brazy J E, Anderson B M, Becker P T, et al. How parents of premature infants gather information and obtain support[J]. Neonatal Netw. 2001, 20(2): 41-48.
[21] Eysenbach G, Kohler C. How do consumers search for and appraise health information on the world wide web? Qualitative study using focus groups, usability tests, and in-depth interviews[J]. BMJ. 2002, 324(7337): 573-577.
[22] Rozic-Hristovsk A, Hristovski D, Todorovski L. Users' information-seeking behavior on a medical library Website[J]. J Med Libr Assoc. 2002, 90(2): 210-217.
[23] Casebeer L, Bennett N, Kristofco R, et al. Physician Internet medical information seeking and on-line continuing education use patterns[J]. J Contin Educ Health Prof. 2002, 22(1): 33-42.
[24] Bessell T L, Silagy C A, Anderson J N, et al. Prevalence of South Australia's online health seekers[J]. Aust N Z J Public Health. 2002, 26(2): 170-173.
[25] Gould M S, Munfakh J L, Lubell K, et al. Seeking help from the internet during adolescence[J]. J Am Acad Child Adolesc Psychiatry. 2002, 41(10): 1182-1189.
[26] Gray N J, Klein J D, Cantrill J A, et al. Adolescent girls' use of the Internet for health information: issues beyond access[J]. J Med Syst. 2002, 26(6): 545-553.
[27] Cullen R J. In search of evidence: family practitioners' use of the Internet for clinical information[J]. J Med Libr Assoc. 2002, 90(4): 370-379.
[28] Fleisher L, Bass S, Ruzek S B, et al. Relationships among Internet health information use, patient behavior and self efficacy in newly diagnosed cancer patients who contact the National Cancer Institute's NCI Atlantic Region Cancer Information Service (CIS)[J]. Proc AMIA Symp. 2002: 260-264.
[29] Frisby G, Bessell T L, Borland R, et al. Smoking cessation and the Internet: a qualitative method examining online consumer behavior[J]. J Med Internet Res. 2002, 4(2): E8.
[30] Menon A M, Deshpande A D, Perri M R, et al. Trust in online prescription drug information among internet users: the impact on information search behavior after exposure to direct-to-consumer advertising[J]. Health Mark Q. 2002, 20(1): 17-35.
[31] Kalichman S C, Benotsch E G, Weinhardt L, et al. Health-related Internet use, coping, social support, and health indicators in people living with HIV/AIDS: preliminary results from a community survey[J]. Health Psychol. 2003, 22(1): 111-116.
[32] De Groote S L, Dorsch J L. Measuring use patterns of online journals and databases[J]. J Med Libr Assoc. 2003, 91(2): 231-240.
[33] Dawes M, Sampson U. Knowledge management in clinical practice: a systematic review of information seeking behavior in physicians[J]. Int J Med Inform. 2003, 71(1): 9-15.
[34] Fujihara N, Miura A. Patterns of searching for information on the World Wide Web: a pilot study[J]. Psychol Rep. 2003, 92(3 Pt 2): 1091-1096.
[35] Huang J Y, Al-Fozan H, Tan S L, et al. Internet use by patients seeking infertility treatment[J]. Int J Gynaecol Obstet. 2003, 83(1): 75-76.
[36] Dutta-Bergman M. Trusted online sources of health information: differences in demographics, health beliefs, and health-information orientation[J]. J Med Internet Res. 2003, 5(3): e21.
[37] Shuyler K S, Knight K M. What are patients seeking when they turn to the Internet?

Qualitative content analysis of questions asked by visitors to an orthopaedics Web site[J]. J Med Internet Res. 2003, 5(4): e24.
[38] Hansen D L, Derry H A, Resnick P J, et al. Adolescents searching for health information on the Internet: an observational study[J]. J Med Internet Res. 2003, 5(4): e25.
[39] Bader J L, Theofanos M F. Searching for cancer information on the internet: analyzing natural language search queries[J]. J Med Internet Res. 2003, 5(4): e31.
[40] Peterson G, Aslani P, Williams K A. How do consumers search for and appraise information on medicines on the Internet? A qualitative study using focus groups[J]. J Med Internet Res. 2003, 5(4): e33.
[41] Stavri P Z, Freeman D J, Burroughs C M. Perception of quality and trustworthiness of Internet resources by personal health information seekers[J]. AMIA Annu Symp Proc. 2003: 629-633.
[42] Adams S. Assessment strategies: how patients cope with the diverse quality levels of websites when searching for health information[J]. AMIA Annu Symp Proc. 2003: 774.
[43] Burk K E, Martin M T, Reilly C A, et al. An online consumer health information resource: 3-year usage summary[J]. AMIA Annu Symp Proc. 2003: 800.
[44] Hanauer D A, Fortin J, Dibble E, et al. Use of the Internet for seeking health care information among young adults[J]. AMIA Annu Symp Proc. 2003: 857.
[45] Tao D, Demiris G, Graves R S, et al. Transition from in library use of resources to outside library use: the impact of the Internet on information seeking behavior of medical students and faculty[J]. AMIA Annu Symp Proc. 2003: 1027.
[46] Bowen D. Predictors of women's Internet access and Internet health seeking[J]. Health Care Women Int. 2003, 24(10): 940-951.
[47] Spink A, Koricich A, Jansen B J, et al. Sexual information seeking on web search engines[J]. Cyberpsychol Behav. 2004, 7(1): 65-72.
[48] Bennett N L, Casebeer L L, Kristofco R E, et al. Physicians' Internet information-seeking behaviors[J]. J Contin Educ Health Prof. 2004, 24(1): 31-38.
[49] Bernhardt J M, Felter E M. Online pediatric information seeking among mothers of young children: results from a qualitative study using focus groups[J]. J Med Internet Res. 2004, 6(1): e7.
[50] Bleakley A, Merzel C R, Vandevanter N L, et al. Computer access and Internet use among urban youths[J]. Am J Public Health. 2004, 94(5): 744-746.
[51] Eysenbach G, Kohler C. Health-related searches on the Internet[J]. JAMA. 2004, 291(24): 2946.
[52] Byrnes J A, Kulick T A, Schwartz D G. Information-seeking behavior changes in community-based teaching practices[J]. J Med Libr Assoc. 2004, 92(3): 334-340.
[53] Dutta-Bergman M J. Health attitudes, health cognitions, and health behaviors among Internet health information seekers: population-based survey[J]. J Med Internet Res. 2004, 6(2): e15.
[54] Dutta-Bergman M J. Primary sources of health information: comparisons in the domain of health attitudes, health cognitions, and health behaviors[J]. Health Commun. 2004, 16(3): 273-288.
[55] Erwin B A, Turk C L, Heimberg R G, et al. The Internet: home to a severe population of individuals with social anxiety disorder?[J]. J Anxiety Disord. 2004, 18(5): 629-646.

[56] Dickerson S, Reinhart A M, Feeley T H, et al. Patient Internet use for health information at three urban primary care clinics[J]. J Am Med Inform Assoc. 2004, 11(6): 499-504.
[57] Ziebland S. The importance of being expert: the quest for cancer information on the Internet[J]. Soc Sci Med. 2004, 59(9): 1783-1793.
[58] Cotten S R, Gupta S S. Characteristics of online and offline health information seekers and factors that discriminate between them[J]. Soc Sci Med. 2004, 59(9): 1795-1806.
[59] Birru M S, Monaco V M, Charles L, et al. Internet usage by low-literacy adults seeking health information: an observational analysis[J]. J Med Internet Res. 2004, 6(3): e25.
[60] Flicker S, Goldberg E, Read S, et al. HIV-positive youth's perspectives on the Internet and e-health[J]. J Med Internet Res. 2004, 6(3): e32.
[61] Grahame M, Laberge J, Scialfa C T. Age differences in search of web pages: the effects of link size, link number, and clutter[J]. Hum Factors. 2004, 46(3): 385-398.
[62] Palesh O, Saltzman K, Koopman C. Internet use and attitudes towards illicit internet use behavior in a sample of Russian college students[J]. Cyberpsychol Behav. 2004, 7(5): 553-558.
[63] Gray N J, Klein J D, Noyce P R, et al. Health information-seeking behaviour in adolescence: the place of the internet[J]. Soc Sci Med. 2005, 60(7): 1467-1478.
[64] Nordfeldt S, Johansson C, Carlsson E, et al. Use of the Internet to search for information in type 1 diabetes children and adolescents: a cross-sectional study[J]. Technol Health Care. 2005, 13(1): 67-74.
[65] Nicholas D, Williams P, Smith A, et al. The information needs of perioperative staff: a preparatory study for a proposed specialist library for theatres (NeLH)[J]. Health Info Libr J. 2005, 22(1): 35-43.
[66] Andrews J E, Pearce K A, Ireson C, et al. Information-seeking behaviors of practitioners in a primary care practice-based research network (PBRN)[J]. J Med Libr Assoc. 2005, 93(2): 206-212.
[67] Ye J. Acculturative stress and use of the Internet among East Asian international students in the United States[J]. Cyberpsychol Behav. 2005, 8(2): 154-161.
[68] Flschman J. Hunting for health: patients are searching for more medical info online[J]. US News World Rep. 2005, 138(20): 46.
[69] Rimer B K, Lyons E J, Ribisl K M, et al. How new subscribers use cancer-related online mailing lists[J]. J Med Internet Res. 2005, 7(3): e32.
[70] Cooper C P, Mallon K P, Leadbetter S, et al. Cancer Internet search activity on a major search engine, United States 2001-2003[J]. J Med Internet Res. 2005, 7(3): e36.
[71] Walji M, Sagaram S, Meric-Bernstam F, et al. Searching for cancer-related information online: unintended retrieval of complementary and alternative medicine information[J]. Int J Med Inform. 2005, 74(7-8): 685-693.
[72] Berger M, Wagner T H, Baker L C. Internet use and stigmatized illness[J]. Soc Sci Med. 2005, 61(8): 1821-1827.
[73] Diaz J A, Sciamanna C N, Evangelou E, et al. Brief report: What types of Internet guidance do patients want from their physicians?[J]. J Gen Intern Med. 2005, 20(8): 683-685.
[74] Kalichman S C, Cain D, Cherry C, et al. Internet use among people living with HIV/AIDS: coping and health-related correlates[J]. AIDS Patient Care STDS. 2005, 19(7): 439-448.

[75] Boissin F G. Information-seeking behaviour and use of the Internet by French general practitioners: a qualitative study[J]. Health Info Libr J. 2005, 22(3): 173-181.
[76] Snipes R L, Ingram R, Jiang P. Information search in health care decision-making: a study of word-of-mouth and internet information users[J]. J Hosp Mark Public Relations. 2005, 15(2): 33-53.
[77] Bright M A, Fleisher L, Thomsen C, et al. Exploring e-Health usage and interest among cancer information service users: the need for personalized interactions and multiple channels remains[J]. J Health Commun. 2005, 10 Suppl 1: 35-52.
[78] Talosig-Garcia M, Davis S W. Information-seeking behavior of minority breast cancer patients: an exploratory study[J]. J Health Commun. 2005, 10 Suppl 1: 53-64.
[79] Rice R E. Influences, usage, and outcomes of Internet health information searching: multivariate results from the Pew surveys[J]. Int J Med Inform. 2006, 75(1): 8-28.
[80] Ybarra M L, Suman M. Help seeking behavior and the Internet: a national survey[J]. Int J Med Inform. 2006, 75(1): 29-41.
[81] Lorence D P, Greenberg L. The zeitgeist of online health search. Implications for a consumer-centric health system[J]. J Gen Intern Med. 2006, 21(2): 134-139.
[82] Dickerson S S, Boehmke M, Ogle C, et al. Seeking and managing hope: patients' experiences using the Internet for cancer care[J]. Oncol Nurs Forum. 2006, 33(1): E8-E17.
[83] Bass S B, Ruzek S B, Gordon T F, et al. Relationship of Internet health information use with patient behavior and self-efficacy: experiences of newly diagnosed cancer patients who contact the National Cancer Institute's Cancer Information Service[J]. J Health Commun. 2006, 11(2): 219-236.
[84] Markman M, Markman M R, Belland A, et al. Profile of ovarian cancer patients seeking information from a web-based decision support program[J]. J Womens Health (Larchmt). 2006, 15(3): 312-318.
[85] Ling B S, Klein W M, Dang Q. Relationship of communication and information measures to colorectal cancer screening utilization: results from HINTS[J]. J Health Commun. 2006, 11 Suppl 1: 181-190.
[86] Bundorf M K, Wagner T H, Singer S J, et al. Who searches the internet for health information?[J]. Health Serv Res. 2006, 41(3 Pt 1): 819-836.
[87] Blake J. Supporting anxious parents in search of Internet information[J]. Nurs Times. 2006, 102(19): 24-26.
[88] Lorence D P, Park H. Measuring dissimilarity in online health search activities[J]. Technol Health Care. 2006, 14(2): 79-89.
[89] Larner A J. Searching the Internet for medical information: frequency over time and by age and gender in an outpatient population in the UK[J]. J Telemed Telecare. 2006, 12(4): 186-188.
[90] Dolinsky C M, Wei S J, Hampshire M K, et al. Breast cancer patients' attitudes toward clinical trials in the radiation oncology clinic versus those searching for trial information on the Internet[J]. Breast J. 2006, 12(4): 324-330.
[91] Schatell D, Wise M, Klicko K, et al. In-center hemodialysis patients' use of the internet in the United States: a national survey[J]. Am J Kidney Dis. 2006, 48(2): 285-291.
[92] Eastin M S, Guinsler N M. Worried and wired: effects of health anxiety on information-seeking and health care utilization behaviors[J]. Cyberpsychol Behav. 2006, 9(4): 494-498.

[93] Renahy E, Chauvin P. Internet uses for health information seeking: A literature review[J]. Rev Epidemiol Sante Publique. 2006, 54(3): 263-275.
[94] Powell J, Clarke A. Internet information-seeking in mental health: population survey[J]. Br J Psychiatry. 2006, 189: 273-277.
[95] Lorence D P, Park H, Fox S. Assessing health consumerism on the Web: a demographic profile of information-seeking behaviors[J]. J Med Syst. 2006, 30(4): 251-258.
[96] Cobb N K, Graham A L. Characterizing Internet searchers of smoking cessation information[J]. J Med Internet Res. 2006, 8(3): e17.
[97] Ahmad F, Hudak P L, Bercovitz K, et al. Are physicians ready for patients with Internet-based health information?[J]. J Med Internet Res. 2006, 8(3): e22.
[98] Lu H Y, Palmgreen P C, Zimmerman R S, et al. Personality traits as predictors of intentions to seek online information about STDs and HIV/AIDS among junior and senior college students in Taiwan[J]. Cyberpsychol Behav. 2006, 9(5): 577-583.
[99] Newnham G M, Burns W I, Snyder R D, et al. Information from the Internet: attitudes of Australian oncology patients[J]. Intern Med J. 2006, 36(11): 718-723.
[100] Stronge A J, Rogers W A, Fisk A D. Web-based information search and retrieval: effects of strategy use and age on search success[J]. Hum Factors. 2006, 48(3): 434-446.
[101] Alpay L, Verhoef J, Toussaint P, et al. What makes an "informed patient"? The impact of contextualization on the search for health information on the Internet[J]. Stud Health Technol Inform. 2006, 124: 913-919.
[102] Delic D, Polasek O, Kern J. Internet health-information seekers in Croatia--who, what, and why?[J]. Med Inform Internet Med. 2006, 31(4): 267-273.
[103] Burkell J A, Wolfe D L, Potter P J, et al. Information needs and information sources of individuals living with spinal cord injury[J]. Health Info Libr J. 2006, 23(4): 257-265.
[104] Grazi G L. Web relationships between physicians and individuals seeking information on hepatopancreatobiliary diseases[J]. Arch Surg. 2006, 141(12): 1176-1182, 1182.
[105] Gaie M J. An evolving user-oriented model of Internet health information seeking[J]. AMIA Annu Symp Proc. 2006: 279-283.
[106] Graham L, Tse T, Keselman A. Exploring user navigation during online health information seeking[J]. AMIA Annu Symp Proc. 2006: 299-303.
[107] Smith C E. Where is it? How deaf adolescents complete fact-based internet search tasks[J]. Am Ann Deaf. 2006, 151(5): 519-529.
[108] Santor D A, Poulin C, Leblanc J C, et al. Online health promotion, early identification of difficulties, and help seeking in young people[J]. J Am Acad Child Adolesc Psychiatry. 2007, 46(1): 50-59.
[109] Tannery N H, Wessel C B, Epstein B A, et al. Hospital nurses' use of knowledge-based information resources[J]. Nurs Outlook. 2007, 55(1): 15-19.
[110] Lorence D, Park H. Study of education disparities and health information seeking behavior[J]. Cyberpsychol Behav. 2007, 10(1): 149-151.
[111] Leung A, Ko P, Chan K S, et al. Searching health information via the web: Hong Kong chinese older adults' experience[J]. Public Health Nurs. 2007, 24(2): 169-175.
[112] Ankem K. Information-seeking behavior of women in their path to an innovative alternate treatment for symptomatic uterine fibroids[J]. J Med Libr Assoc. 2007, 95(2): 164-172, e51-e53.

[113] Volk R M. Expert searching in consumer health: an important role for librarians in the age of the Internet and the Web[J]. J Med Libr Assoc. 2007, 95(2): 203-207, e66.
[114] Porter A, Edirippulige S. Parents of deaf children seeking hearing loss-related information on the internet: the Australian experience[J]. J Deaf Stud Deaf Educ. 2007, 12(4): 518-529.
[115] Davies K, Harrison J. The information-seeking behaviour of doctors: a review of the evidence[J]. Health Info Libr J. 2007, 24(2): 78-94.
[116] Wu J, Gipson T, Chin N, et al. Women seeking emergency contraceptive pills by using the internet[J]. Obstet Gynecol. 2007, 110(1): 44-52.
[117] Ayers S L, Kronenfeld J J. Chronic illness and health-seeking information on the Internet[J]. Health (London). 2007, 11(3): 327-347.
[118] Singh P M, Wight C A, Sercinoglu O, et al. Language preferences on websites and in Google searches for human health and food information[J]. J Med Internet Res. 2007, 9(2): e18.
[119] Phua J, Lim T K. Use of traditional versus electronic medical-information resources by residents and interns[J]. Med Teach. 2007, 29(4): 400-402.
[120] Rahmqvist M, Bara A C. Patients retrieving additional information via the Internet: a trend analysis in a Swedish population, 2000-05[J]. Scand J Public Health. 2007, 35(5): 533-539.
[121] Buntrock S, Hopfgarten T, Adolfsson J, et al. The Internet and prostate cancer patients: searching for and finding information[J]. Scand J Urol Nephrol. 2007, 41(5): 367-374.
[122] Muller H, Boyer C, Gaudinat A, et al. Analyzing web log files of the health on the net HONmedia search engine to define typical image search tasks for image retrieval evaluation[J]. Stud Health Technol Inform. 2007, 129(Pt 2): 1319-1323.
[123] Lin C C, Tsai C C. A navigation flow map method of representing students' searching behaviors and strategies on the web, with relation to searching outcomes[J]. Cyberpsychol Behav. 2007, 10(5): 689-695.
[124] Lu H Y, Case D O, Lustria M L, et al. Predictors of online information seeking by international students when disaster strikes their countries[J]. Cyberpsychol Behav. 2007, 10(5): 709-712.
[125] Rains S A. Perceptions of traditional information sources and use of the world wide web to seek health information: findings from the health information national trends survey[J]. J Health Commun. 2007, 12(7): 667-680.
[126] Kim S, Chung D S. Characteristics of cancer blog users[J]. J Med Libr Assoc. 2007, 95(4): 445-450.
[127] Warner D, Procaccino J D. Women seeking health information: distinguishing the web user[J]. J Health Commun. 2007, 12(8): 787-814.
[128] Deluca P, Schifano F. Searching the Internet for drug-related web sites: analysis of online available information on ecstasy (MDMA)[J]. Am J Addict. 2007, 16(6): 479-483.
[129] Johnson G M, Kulpa A. Dimensions of online behavior: toward a user typology[J]. Cyberpsychol Behav. 2007, 10(6): 773-779.
[130] Nwagwu W E. The Internet as a source of reproductive health information among adolescent girls in an urban city in Nigeria[J]. BMC Public Health. 2007, 7: 354.
[131] Guiding parents in their search for high-quality health information on the Internet[J].

Paediatr Child Health. 2007, 12(3): 239-240.
[132] Dey A, Reid B, Godding R, et al. Perceptions and behaviour of access of the Internet: a study of women attending a breast screening service in Sydney, Australia[J]. Int J Med Inform. 2008, 77(1): 24-32.
[133] Munoz-Izquierdo A, Puchades-Simo A, Marco-Gisbert A, et al.[Access to the internet among nurses and type of information sought][J]. Enferm Clin. 2008, 18(1): 18-25.
[134] Renahy E, Parizot I, Chauvin P. Health information seeking on the Internet: a double divide? Results from a representative survey in the Paris metropolitan area, France, 2005-2006[J]. BMC Public Health. 2008, 8: 69.
[135] Neelapala P, Duvvi S K, Kumar G, et al. Do gynaecology outpatients use the Internet to seek health information? A questionnaire survey[J]. J Eval Clin Pract. 2008, 14(2): 300-304.
[136] Tian Y, Robinson J D. Incidental health information use and media complementarity: a comparison of senior and non-senior cancer patients[J]. Patient Educ Couns. 2008, 71(3): 340-344.
[137] Pena-Purcell N. Hispanics' use of Internet health information: an exploratory study[J]. J Med Libr Assoc. 2008, 96(2): 101-107.
[138] Hay M C, Cadigan R J, Khanna D, et al. Prepared patients: internet information seeking by new rheumatology patients[J]. Arthritis Rheum. 2008, 59(4): 575-582.
[139] Tian Y, Robinson J D. Media use and health information seeking: an empirical test of complementarity theory[J]. Health Commun. 2008, 23(2): 184-190.
[140] Shin D H, Kim W Y. Applying the Technology Acceptance Model and flow theory to Cyworld user behavior: implication of the Web2.0 user acceptance[J]. Cyberpsychol Behav. 2008, 11(3): 378-382.
[141] Khoo K, Bolt P, Babl F E, et al. Health information seeking by parents in the Internet age[J]. J Paediatr Child Health. 2008, 44(7-8): 419-423.
[142] Shaw B R, Dubenske L L, Han J Y, et al. Antecedent characteristics of online cancer information seeking among rural breast cancer patients: an application of the Cognitive-Social Health Information Processing (C-SHIP) model[J]. J Health Commun. 2008, 13(4): 389-408.
[143] Hausner H, Hajak G, Spiessl H. Gender differences in help-seeking behavior on two internet forums for individuals with self-reported depression[J]. Gend Med. 2008, 5(2): 181-185.
[144] Hughes S, Dennison C R. Progress in prevention: how can we help patients seek information on the World Wide Web?: an opportunity to improve the "net effect"[J]. J Cardiovasc Nurs. 2008, 23(4): 324-325.
[145] Gorrindo T, Groves J E. Web searching for information about physicians[J]. JAMA. 2008, 300(2): 213-215.
[146] Spallek H, Butler B S, Schleyer T K, et al. Supporting emerging disciplines with e-communities: needs and benefits[J]. J Med Internet Res. 2008, 10(2): e19.
[147] Tuil W S, Verhaak C M, De Vries R P, et al. IVF patients show three types of online behaviour[J]. Hum Reprod. 2008, 23(11): 2501-2505.
[148] Lee C J. Does the internet displace health professionals?[J]. J Health Commun. 2008, 13(5): 450-464.
[149] Bouche G, Migeot V. Parental use of the Internet to seek health information and primary care utilisation for their child: a cross-sectional study[J]. BMC Public Health. 2008,

8: 300.
[150] Khazaal Y, Chatton A, Cochand S, et al. Internet use by patients with psychiatric disorders in search for general and medical informations[J]. Psychiatr Q. 2008, 79(4): 301-309.
[151] Leung L. Internet embeddedness: links with online health information seeking, expectancy value/quality of health information websites, and Internet usage patterns[J]. Cyberpsychol Behav. 2008, 11(5): 565-569.
[152] Kovic I, Lulic I, Brumini G. Examining the medical blogosphere: an online survey of medical bloggers[J]. J Med Internet Res. 2008, 10(3): e28.
[153] Cain D S. Parenting online and lay literature on infant spanking: information readily available to parents[J]. Soc Work Health Care. 2008, 47(2): 174-184.
[154] Abrahamson J A, Fisher K E, Turner A G, et al. Lay information mediary behavior uncovered: exploring how nonprofessionals seek health information for themselves and others online[J]. J Med Libr Assoc. 2008, 96(4): 310-323.
[155] Hong Y, Gillis R D, Donnell R F. Use of consumer health vocabularies in online physician directory to improve physician search[J]. AMIA Annu Symp Proc. 2008: 974.
[156] Mahoui M, Jones J, Zollinger D, et al. Leveraging user search behavior to design personalized browsing interfaces for healthcare Web sites[J]. AMIA Annu Symp Proc. 2008: 994.
[157] Hunscher D A. The evolving state of online search for consumer health information[J]. AMIA Annu Symp Proc. 2008: 983.
[158] Hay M C, Strathmann C, Lieber E, et al. Why patients go online: multiple sclerosis, the internet, and physician-patient communication[J]. Neurologist. 2008, 14(6): 374-381.
[159] Mcmillan S J, Macias W. Strengthening the safety net for online seniors: factors influencing differences in health information seeking among older internet users[J]. J Health Commun. 2008, 13(8): 778-792.
[160] Larsson M. A descriptive study of the use of the Internet by women seeking pregnancy-related information[J]. Midwifery. 2009, 25(1): 14-20.
[161] Schielein T, Klein H E, Hubner-Liebermann B, et al.[Via internet to the psychiatrist. The internet as a first step to seek psychiatric advice][J]. Psychiatr Prax. 2009, 36(1): 40-42.
[162] Roche M I, Skinner D. How parents search, interpret, and evaluate genetic information obtained from the internet[J]. J Genet Couns. 2009, 18(2): 119-129.
[163] Turner A M, Petrochilos D, Nelson D E, et al. Access and use of the Internet for health information seeking: a survey of local public health professionals in the northwest[J]. J Public Health Manag Pract. 2009, 15(1): 67-69.
[164] Bylund C L, Gueguen J A, D'Agostino T A, et al. Cancer patients' decisions about discussing Internet information with their doctors[J]. Psychooncology. 2009, 18(11): 1139-1146.
[165] Small G W, Moody T D, Siddarth P, et al. Your brain on Google: patterns of cerebral activation during internet searching[J]. Am J Geriatr Psychiatry. 2009, 17(2): 116-126.
[166] Noh H I, Lee J M, Yun Y H, et al. Cervical cancer patient information-seeking behaviors, information needs, and information sources in South Korea[J]. Support Care Cancer. 2009, 17(10): 1277-1283.
[167] Lee C J, Hornik R C. Physician trust moderates the Internet use and physician visit

relationship[J]. J Health Commun. 2009, 14(1): 70-76.
[168] Kivits J. Everyday health and the internet: a mediated health perspective on health information seeking[J]. Sociol Health Illn. 2009, 31(5): 673-687.
[169] Atkinson N L, Saperstein S L, Pleis J. Using the internet for health-related activities: findings from a national probability sample[J]. J Med Internet Res. 2009, 11(1): e4.
[170] Lee Y J, Park J, Widdows R. Exploring antecedents of consumer satisfaction and repeated search behavior on e-health information[J]. J Health Commun. 2009, 14(2): 160-173.
[171] Madle G, Berger A, Cognat S, et al. User information seeking behaviour: perceptions and reality. An evaluation of the WHO Labresources Internet portal[J]. Inform Health Soc Care. 2009, 34(1): 30-38.
[172] Ortego C N, Barnosi M A, Simeon A C, et al.[Search for information on the Internet by patients with chronic autoimmune diseases in different Spanish populations][J]. Med Clin (Barc). 2009, 133(12): 467-471.
[173] Laurent M R, Vickers T J. Seeking health information online: does Wikipedia matter?[J]. J Am Med Inform Assoc. 2009, 16(4): 471-479.
[174] Akhu-Zaheya L M, Dickerson S S. Jordanian patients' and caregivers' use of the internet for seeking health information[J]. Comput Inform Nurs. 2009, 27(3): 184-191.
[175] Zhang Y, Jones B, Spalding M, et al. Use of the internet for health information among primary care patients in rural West Texas[J]. South Med J. 2009, 102(6): 595-601.
[176] Prendiville T W, Saunders J, Fitzsimons J. The information-seeking behaviour of paediatricians accessing web-based resources[J]. Arch Dis Child. 2009, 94(8): 633-635.
[177] Hughes B, Joshi I, Lemonde H, et al. Junior physician's use of Web 2.0 for information seeking and medical education: a qualitative study[J]. Int J Med Inform. 2009, 78(10): 645-655.
[178] Weaver J R, Mays D, Lindner G, et al. Profiling characteristics of internet medical information users[J]. J Am Med Inform Assoc. 2009, 16(5): 714-722.
[179] Navigating the ocean of health information. Tips for searching the Internet for information about heart disease[J]. Harv Heart Lett. 2009, 19(5): 6.
[180] Verhoeven V, Baay M, Baay P. People seeking health information about human papillomavirus via the internet have a very high level of anxiety[J]. Sex Health. 2009, 6(3): 258-259.
[181] Shute N. Connecting and sharing on the Web. At 'crowd-sourced' disease sites, patients can swap stories-and data[J]. US News World Rep. 2009, 146(7): 82-83.
[182] Borzekowski D L, Leith J, Medoff D R, et al. Use of the internet and other media for health information among clinic outpatients with serious mental illness[J]. Psychiatr Serv. 2009, 60(9): 1265-1268.
[183] Xie B. Older adults' health information wants in the internet age: implications for patient-provider relationships[J]. J Health Commun. 2009, 14(6): 510-524.
[184] Kelly K M, Sturm A C, Kemp K, et al. How can we reach them? Information seeking and preferences for a cancer family history campaign in underserved communities[J]. J Health Commun. 2009, 14(6): 573-589.
[185] Taha J, Sharit J, Czaja S. Use of and satisfaction with sources of health information among older Internet users and nonusers[J]. Gerontologist. 2009, 49(5): 663-673.
[186] Zhao S. Parental education and children's online health information seeking: beyond the

digital divide debate[J]. Soc Sci Med. 2009, 69(10): 1501-1505.
[187] Tsitsika A, Critselis E, Kormas G, et al. Adolescent pornographic internet site use: a multivariate regression analysis of the predictive factors of use and psychosocial implications[J]. Cyberpsychol Behav. 2009, 12(5): 545-550.
[188] Shieh C, Mays R, Mcdaniel A, et al. Health literacy and its association with the use of information sources and with barriers to information seeking in clinic-based pregnant women[J]. Health Care Women Int. 2009, 30(11): 971-988.
[189] Buhi E R, Daley E M, Fuhrmann H J, et al. An observational study of how young people search for online sexual health information[J]. J Am Coll Health. 2009, 58(2): 101-111.
[190] Wong F W, Lin L, Lim D C. Drug and herb interactions: searching the web[J]. Aust Fam Physician. 2009, 38(8): 627-633.
[191] Kitikannakorn N, Sitthiworanan C. Searching for health information on the Internet by undergraduate students in Phitsanulok, Thailand[J]. Int J Adolesc Med Health. 2009, 21(3): 313-318.
[192] Horvath K J, Courtenay-Quirk C, Harwood E, et al. Using the Internet to provide care for persons living with HIV[J]. AIDS Patient Care STDS. 2009, 23(12): 1033-1041.
[193] Lobach D F, Waters A, Silvey G M, et al. Facilitating consumer clinical information seeking by maintaining referential context: evaluation of a prototypic approach[J]. AMIA Annu Symp Proc. 2009, 2009: 380-384.
[194] White R W, Horvitz E. Experiences with web search on medical concerns and self diagnosis[J]. AMIA Annu Symp Proc. 2009, 2009: 696-700.
[195] Miller R. Web portals and patient information-seeking behaviors[J]. J Oncol Pract. 2009, 5(4): 182-183.
[196] Gallagher S, Doherty D T. Searching for health information online: characteristics of online health seekers[J]. J Evid Based Med. 2009, 2(2): 99-106.
[197] Shinchuk L M, Chiou P, Czarnowski V, et al. Demographics and attitudes of chronic-pain patients who seek online pain-related medical information: implications for healthcare providers[J]. Am J Phys Med Rehabil. 2010, 89(2): 141-146.
[198] Mccann A L, Schneiderman E D, Hinton R J. E-teaching and learning preferences of dental and dental hygiene students[J]. J Dent Educ. 2010, 74(1): 65-78.
[199] Baxter R. Exponential growth using the internet and your web site[J]. Facial Plast Surg. 2010, 26(1): 39-44.
[200] Chisolm D J. Does online health information seeking act like a health behavior?: a test of the behavioral model[J]. Telemed J E Health. 2010, 16(2): 154-160.
[201] Gomella L G. Doctor google and the internet prescription[J]. Can J Urol. 2010, 17(1): 4971.
[202] Lam-Po-Tang J, Mckay D. Dr Google, MD: a survey of mental health-related internet use in a private practice sample[J]. Australas Psychiatry. 2010, 18(2): 130-133.
[203] Cobb N K. Online consumer search strategies for smoking-cessation information[J]. Am J Prev Med. 2010, 38(3 Suppl): S429-S432.
[204] Reavley N, Jorm A, Morgan A, et al. Mental health information on the Internet: a new wiki guide[J]. Aust N Z J Psychiatry. 2010, 44(3): 291.
[205] Hesse B W, Moser R P, Rutten L J. Surveys of physicians and electronic health

information[J]. N Engl J Med. 2010, 362(9): 859-860.
[206] Corcoran T B, Haigh F, Seabrook A, et al. A survey of patients' use of the internet for chronic pain-related information[J]. Pain Med. 2010, 11(4): 512-517.
[207] Overberg R, Otten W, de Man A, et al. How breast cancer patients want to search for and retrieve information from stories of other patients on the internet: an online randomized controlled experiment[J]. J Med Internet Res. 2010, 12(1): e7.
[208] Clinton B K, Silverman B C, Brendel D H. Patient-targeted googling: the ethics of searching online for patient information[J]. Harv Rev Psychiatry. 2010, 18(2): 103-112.
[209] Renahy E, Parizot I, Chauvin P. Determinants of the frequency of online health information seeking: results of a web-based survey conducted in France in 2007[J]. Inform Health Soc Care. 2010, 35(1): 25-39.
[210] Tustin N. The role of patient satisfaction in online health information seeking[J]. J Health Commun. 2010, 15(1): 3-17.
[211] Lee S Y, Hawkins R. Why do patients seek an alternative channel? The effects of unmet needs on patients' health-related Internet use[J]. J Health Commun. 2010, 15(2): 152-166.
[212] Younger P. Internet-based information-seeking behaviour amongst doctors and nurses: a short review of the literature[J]. Health Info Libr J. 2010, 27(1): 2-10.
[213] Verhoeven F, Steehouder M F, Hendrix R M, et al. How nurses seek and evaluate clinical guidelines on the Internet[J]. J Adv Nurs. 2010, 66(1): 114-127.
[214] Coberly E, Boren S A, Davis J W, et al. Linking clinic patients to Internet-based, condition-specific information prescriptions[J]. J Med Libr Assoc. 2010, 98(2): 160-164.
[215] Yan Y Y. Online health information seeking behavior in Hong Kong: an exploratory study[J]. J Med Syst. 2010, 34(2): 147-153.
[216] Caiata-Zufferey M, Abraham A, Sommerhalder K, et al. Online health information seeking in the context of the medical consultation in Switzerland[J]. Qual Health Res. 2010, 20(8): 1050-1061.
[217] De Santis M, De Luca C, Quattrocchi T, et al. Use of the Internet by women seeking information about potentially teratogenic agents[J]. Eur J Obstet Gynecol Reprod Biol. 2010, 151(2): 154-157.
[218] Cutilli C C. Seeking health information: what sources do your patients use?[J]. Orthop Nurs. 2010, 29(3): 214-219.
[219] Nalliah S, Chan S L, Ong C L, et al. Effectiveness of the use of internet search by third year medical students to establish a clinical diagnosis[J]. Singapore Med J. 2010, 51(4): 332-338.
[220] Dommes A, Chevalier A, Rossetti M. Searching for information on the World Wide Web with a search engine: a pilot study on cognitive flexibility in younger and older users[J]. Psychol Rep. 2010, 106(2): 490-498.
[221] Breyer B N, Eisenberg M L. Use of Google in study of noninfectious medical conditions[J]. Epidemiology. 2010, 21(4): 584-585.
[222] Lagan B M, Sinclair M, Kernohan W G. Internet use in pregnancy informs women's decision making: a web-based survey[J]. Birth. 2010, 37(2): 106-115.
[223] Weaver J R, Mays D, Weaver S S, et al. Health information-seeking behaviors, health indicators, and health risks[J]. Am J Public Health. 2010, 100(8): 1520-1525.
[224] Boot C R, Meijman F J. The public and the Internet: multifaceted drives for seeking

health information[J]. Health Informatics J. 2010, 16(2): 145-156.
[225] Wacogne I, Scott-Jupp R. The role of Google in children's health[J]. Arch Dis Child. 2010, 95(8): 576-577.
[226] Walsh M C, Trentham-Dietz A, Schroepfer T A, et al. Cancer information sources used by patients to inform and influence treatment decisions[J]. J Health Commun. 2010, 15(4): 445-463.
[227] Simmat-Durand L. Pregnancy under subutex (buprenorphine): opinions expressed on French internet forums[J]. Subst Use Misuse. 2010, 45(10): 1652-1667.
[228] Yun E K, Park H A. Consumers' disease information-seeking behaviour on the Internet in Korea[J]. J Clin Nurs. 2010, 19(19-20): 2860-2868.
[229] Reinfeld-Kirkman N, Kalucy E, Roeger L. The relationship between self-reported health status and the increasing likelihood of South Australians seeking Internet health information[J]. Aust N Z J Public Health. 2010, 34(4): 422-426.
[230] Kernisan L P, Sudore R L, Knight S J. Information-seeking at a caregiving website: a qualitative analysis[J]. J Med Internet Res. 2010, 12(3): e31.
[231] Edson R S, Beckman T J, West C P, et al. A multi-institutional survey of internal medicine residents' learning habits[J]. Med Teach. 2010, 32(9): 773-775.
[232] Wilkerson J M, Smolenski D J, Horvath K J, et al. Online and offline sexual health-seeking patterns of HIV-negative men who have sex with men[J]. AIDS Behav. 2010, 14(6): 1362-1370.
[233] Lubowitz J H, Poehling G G. Information overload: technology, the internet, and arthroscopy[J]. Arthroscopy. 2010, 26(9): 1141-1143.
[234] Yardley L, Morrison L G, Andreou P, et al. Understanding reactions to an internet-delivered health-care intervention: accommodating user preferences for information provision[J]. BMC Med Inform Decis Mak. 2010, 10: 52.
[235] Fu K W, Wong P W, Yip P S. What do internet users seek to know about depression from web searches? A descriptive study of 21 million web queries[J]. J Clin Psychiatry. 2010, 71(9): 1246-1247.
[236] Nagler R H, Gray S W, Romantan A, et al. Differences in information seeking among breast, prostate, and colorectal cancer patients: results from a population-based survey[J]. Patient Educ Couns. 2010, 81 Suppl: S54-S62.
[237] Boyer C. Education and consumer informatics[J]. Yearb Med Inform. 2010: 72-74.
[238] Wise K, Alhabash S, Park H. Emotional responses during social information seeking on Facebook[J]. Cyberpsychol Behav Soc Netw. 2010, 13(5): 555-562.
[239] Kim K, Kwon N. Profile of e-patients: analysis of their cancer information-seeking from a national survey[J]. J Health Commun. 2010, 15(7): 712-733.
[240] Kelly B, Hornik R, Romantan A, et al. Cancer information scanning and seeking in the general population[J]. J Health Commun. 2010, 15(7): 734-753.
[241] Fogel J, Fajiram S, Morgan P D. Sexual health information seeking on the Internet: comparisons between White and African American college students[J]. ABNF J. 2010, 21(4): 79-84.
[242] Tortolero-Luna G, Finney R L, Hesse B W, et al. Health and cancer information seeking practices and preferences in Puerto Rico: creating an evidence base for cancer communication efforts[J]. J Health Commun. 2010, 15 Suppl 3: 30-45.

[243] Hou J, Shim M. The role of provider-patient communication and trust in online sources in Internet use for health-related activities[J]. J Health Commun. 2010, 15 Suppl 3: 186-199.
[244] Ye Y. A path analysis on correlates of consumer trust in online health information: evidence from the health information national trends survey[J]. J Health Commun. 2010, 15 Suppl 3: 200-215.
[245] Kealey E, Berkman C S. The relationship between health information sources and mental models of cancer: findings from the 2005 Health Information National Trends Survey[J]. J Health Commun. 2010, 15 Suppl 3: 236-251.
[246] Koch-Weser S, Bradshaw Y S, Gualtieri L, et al. The Internet as a health information source: findings from the 2007 Health Information National Trends Survey and implications for health communication[J]. J Health Commun. 2010, 15 Suppl 3: 279-293.
[247] Klein B, White A, Kavanagh D, et al. Content and functionality of alcohol and other drug websites: results of an online survey[J]. J Med Internet Res. 2010, 12(5): e51.
[248] Schrank B, Sibitz I, Unger A, et al. How patients with schizophrenia use the internet: qualitative study[J]. J Med Internet Res. 2010, 12(5): e70.
[249] Patel C O, Garg V, Khan S A. What do patients search for when seeking clinical trial information online?[J]. AMIA Annu Symp Proc. 2010, 2010: 597-601.
[250] Rorat M, Kuchar E, Szenborn L, et al.[Growing boreliosis anxiety and its reasons][J]. Psychiatr Pol. 2010, 44(6): 895-904.
[251] Holmberg C, Harttig U, Schulze M B, et al. The potential of the Internet for health communication: the use of an interactive on-line tool for diabetes risk prediction[J]. Patient Educ Couns. 2011, 83(1): 106-112.
[252] Dickerson S S, Reinhart A, Boemhke M, et al. Cancer as a problem to be solved: internet use and provider communication by men with cancer[J]. Comput Inform Nurs. 2011, 29(7): 388-395.
[253] Ye Y. Correlates of consumer trust in online health information: findings from the health information national trends survey[J]. J Health Commun. 2011, 16(1): 34-49.
[254] Lewis S, Thomas S L, Blood R W, et al. 'I'm searching for solutions': why are obese individuals turning to the Internet for help and support with 'being fat'?[J]. Health Expect. 2011, 14(4): 339-350.
[255] Jones R K, Biddlecom A E. Is the internet filling the sexual health information gap for teens? An exploratory study[J]. J Health Commun. 2011, 16(2): 112-123.
[256] Han J Y. Transaction logfile analysis in health communication research: challenges and opportunities[J]. Patient Educ Couns. 2011, 82(3): 307-312.
[257] Bell R A, Hu X, Orrange S E, et al. Lingering questions and doubts: online information-seeking of support forum members following their medical visits[J]. Patient Educ Couns. 2011, 85(3): 525-528.
[258] Zulman D M, Kirch M, Zheng K, et al. Trust in the internet as a health resource among older adults: analysis of data from a nationally representative survey[J]. J Med Internet Res. 2011, 13(1): e19.
[259] Alghamdi K M, Almohideb M A. Internet use by dermatology outpatients to search for health information[J]. Int J Dermatol. 2011, 50(3): 292-299.
[260] Powell J, Inglis N, Ronnie J, et al. The characteristics and motivations of online health information seekers: cross-sectional survey and qualitative interview study[J]. J Med Internet

Res. 2011, 13(1): e20.
[261] Shariff S Z, Bejaimal S A, Sontrop J M, et al. Searching for medical information online: a survey of Canadian nephrologists[J]. J Nephrol. 2011, 24(6): 723-732.
[262] Knapp C, Madden V, Marcu M, et al. Information seeking behaviors of parents whose children have life-threatening illnesses[J]. Pediatr Blood Cancer. 2011, 56(5): 805-811.
[263] Yang A C, Tsai S J, Huang N E, et al. Association of Internet search trends with suicide death in Taipei City, Taiwan, 2004-2009[J]. J Affect Disord. 2011, 132(1-2): 179-184.
[264] Wang J Y, Bennett K, Probst J. Subdividing the digital divide: differences in internet access and use among rural residents with medical limitations[J]. J Med Internet Res. 2011, 13(1): e25.
[265] Brooks-Pollock E, Tilston N, Edmunds W J, et al. Using an online survey of healthcare-seeking behaviour to estimate the magnitude and severity of the 2009 H1N1v influenza epidemic in England[J]. BMC Infect Dis. 2011, 11: 68.
[266] Neal D M, Mckenzie P J. Putting the pieces together: endometriosis blogs, cognitive authority, and collaborative information behavior[J]. J Med Libr Assoc. 2011, 99(2): 127-134.
[267] Nambisan P. Information seeking and social support in online health communities: impact on patients' perceived empathy[J]. J Am Med Inform Assoc. 2011, 18(3): 298-304.
[268] Santana S, Lausen B, Bujnowska-Fedak M, et al. Informed citizen and empowered citizen in health: results from an European survey[J]. BMC Fam Pract. 2011, 12: 20.
[269] Percheski C, Hargittai E. Health information-seeking in the digital age[J]. J Am Coll Health. 2011, 59(5): 379-386.
[270] Kuehn B M. Patients go online seeking support, practical advice on health conditions[J]. JAMA. 2011, 305(16): 1644-1645.
[271] Dolce M C. The Internet as a source of health information: experiences of cancer survivors and caregivers with healthcare providers[J]. Oncol Nurs Forum. 2011, 38(3): 353-359.
[272] van Deursen A J, van Dijk J A. Internet skills performance tests: are people ready for eHealth?[J]. J Med Internet Res. 2011, 13(2): e35.
[273] Kishimoto K, Fukushima N. Use of anonymous Web communities and websites by medical consumers in Japan to research drug information[J]. Yakugaku Zasshi. 2011, 131(5): 685-695.
[274] Cohall A T, Nye A, Moon-Howard J, et al. Computer use, internet access, and online health searching among Harlem adults[J]. Am J Health Promot. 2011, 25(5): 325-333.
[275] Baumgartner S E, Hartmann T. The role of health anxiety in online health information search[J]. Cyberpsychol Behav Soc Netw. 2011, 14(10): 613-618.
[276] Hill S, Mao J, Ungar L, et al. Natural supplements for H1N1 influenza: retrospective observational infodemiology study of information and search activity on the Internet[J]. J Med Internet Res. 2011, 13(2): e36.
[277] Brunsman-Johnson C, Narayanan S, Shebilske W, et al. Modeling web-based information seeking by users who are blind[J]. Disabil Rehabil Assist Technol. 2011, 6(6): 511-525.
[278] Becze E. Survivors seeking cancer information online can experience disenchantment, empowerment[J]. ONS Connect. 2011, 26(4): 14-15.
[279] Samal L, Saha S, Chander G, et al. Internet health information seeking behavior and

antiretroviral adherence in persons living with HIV/AIDS[J]. AIDS Patient Care STDS. 2011, 25(7): 445-449.
[280] Pourmand A, Sikka N. Online health information impacts patients' decisions to seek emergency department care[J]. West J Emerg Med. 2011, 12(2): 174-177.
[281] Robins P. Yes, skin cancer consumer education is just a click away[J]. Dermatol Surg. 2011, 37(7): 1065-1066.
[282] Choi N. Relationship between health service use and health information technology use among older adults: analysis of the US National Health Interview Survey[J]. J Med Internet Res. 2011, 13(2): e33.
[283] Ben-Sasson A. Parents' search for evidence-based practice: a personal story[J]. J Paediatr Child Health. 2011, 47(7): 415-418.
[284] Rapple B.[When patients use internet education][J]. Krankenpfl Soins Infirm. 2011, 104(7): 20-23.
[285] Weiner S A, Stephens G, Nour A Y. Information-seeking behaviors of first-semester veterinary students: a preliminary report[J]. J Vet Med Educ. 2011, 38(1): 21-32.
[286] Chang S S, Page A, Gunnell D. Internet searches for a specific suicide method follow its high-profile media coverage[J]. Am J Psychiatry. 2011, 168(8): 855-857.
[287] Cowie G A, Hill S, Robinson P. Using an online service for breastfeeding support: what mothers want to discuss[J]. Health Promot J Austr. 2011, 22(2): 113-118.
[288] Kim H, Park S Y, Bozeman I. Online health information search and evaluation: observations and semi-structured interviews with college students and maternal health experts[J]. Health Info Libr J. 2011, 28(3): 188-199.
[289] Klinar I, Balazin A, Barsic B, et al. Identification of general characteristics, motivation, and satisfaction of internet-based medical consultation service users in Croatia[J]. Croat Med J. 2011, 52(4): 557-565.
[290] Yuen H K, Azuero A, London S. Association between seeking oral health information online and knowledge in adults with spinal cord injury: a pilot study[J]. J Spinal Cord Med. 2011, 34(4): 423-431.
[291] Liang H, Xue Y, Chase S K. Online health information seeking by people with physical disabilities due to neurological conditions[J]. Int J Med Inform. 2011, 80(11): 745-753.
[292] Boyer C. Consumer informatics: control or making the most of health internet websites[J]. Yearb Med Inform. 2011, 6(1): 142-145.
[293] Laz T H, Berenson A B. Association of web-based weight loss information use with weight reduction behaviors in adolescent women[J]. J Adolesc Health. 2011, 49(4): 446-448.
[294] Lim S, Xue L, Yen C C, et al. A study on Singaporean women's acceptance of using mobile phones to seek health information[J]. Int J Med Inform. 2011, 80(12): e189-e202.
[295] Glynn R W, Kelly J C, Coffey N, et al. The effect of breast cancer awareness month on internet search activity--a comparison with awareness campaigns for lung and prostate cancer[J]. BMC Cancer. 2011, 11: 442.
[296] Shabi I N, Shabi O M, Akewukereke M A, et al. Physicians utilisation of internet medical databases at the tertiary health institutions in Osun State, south west, Nigeria[J]. Health Info Libr J. 2011, 28(4): 313-320.
[297] Liu Y, Farris K B, Doucette W R. Factors affecting osteoarthritis patients' self-reported goal-directed drug information-seeking behaviors after exposure to direct-to-consumer

advertising from physicians and the internet[J]. Hosp Pract (1995). 2011, 39(4): 76-81.
[298] Robertson-Lang L, Major S, Hemming H. An exploration of search patterns and credibility issues among older adults seeking online health information[J]. Can J Aging. 2011, 30(4): 631-645.
[299] Lagan B M, Sinclair M, Kernohan W G. What is the impact of the Internet on decision-making in pregnancy? A global study[J]. Birth. 2011, 38(4): 336-345.
[300] Roehr B. Trend for US patients to seek health information from media and internet is stalling[J]. BMJ. 2011, 343: d7738.
[301] Takahashi Y, Ohura T, Ishizaki T, et al. Internet use for health-related information via personal computers and cell phones in Japan: a cross-sectional population-based survey[J]. J Med Internet Res. 2011, 13(4): e110.
[302] Paul C L, Carey M L, Hall A E, et al. Improving access to information and support for patients with less common cancers: hematologic cancer patients' views about Web-based approaches[J]. J Med Internet Res. 2011, 13(4): e112.
[303] Barman-Adhikari A, Rice E. Sexual Health Information Seeking Online Among Runaway and Homeless Youth[J]. J Soc Social Work Res. 2011, 2(2): 88-103.
[304] Ludgate M W, Sabel M S, Fullen D R, et al. Internet use and anxiety in people with melanoma and nonmelanoma skin cancer[J]. Dermatol Surg. 2011, 37(9): 1252-1259.
[305] Shaw R J, Johnson C M. Health Information Seeking and Social Media Use on the Internet among People with Diabetes[J]. Online J Public Health Inform. 2011, 3(1).
[306] Magee J C, Bigelow L, Dehaan S, et al. Sexual health information seeking online: a mixed-methods study among lesbian, gay, bisexual, and transgender young people[J]. Health Educ Behav. 2012, 39(3): 276-289.
[307] Simone C N, Hampshire M K, Vachani C, et al. The utilization of oncology web-based resources in Spanish-speaking Internet users[J]. Am J Clin Oncol. 2012, 35(6): 520-526.
[308] Muusses L D, van Weert J C, van Dulmen S, et al. Chemotherapy and information-seeking behaviour: characteristics of patients using mass-media information sources[J]. Psychooncology. 2012, 21(9): 993-1002.
[309] O'Leary D F, Mhaolrunaigh S N. Information-seeking behaviour of nurses: where is information sought and what processes are followed?[J]. J Adv Nurs. 2012, 68(2): 379-390.
[310] Weeks B E, Friedenberg L M, Southwell B G, et al. Behavioral consequences of conflict-oriented health news coverage: the 2009 mammography guideline controversy and online information seeking[J]. Health Commun. 2012, 27(2): 158-166.
[311] Tausczik Y, Faasse K, Pennebaker J W, et al. Public anxiety and information seeking following the H1N1 outbreak: blogs, newspaper articles, and Wikipedia visits[J]. Health Commun. 2012, 27(2): 179-185.
[312] Karras E, Rintamaki L S. An examination of online health information seeking by deaf people[J]. Health Commun. 2012, 27(2): 194-204.
[313] Gage E A, Panagakis C. The devil you know: parents seeking information online for paediatric cancer[J]. Sociol Health Illn. 2012, 34(3): 444-458.
[314] Kassan E C, Williams R M, Kelly S P, et al. Men's use of an Internet-based decision aid for prostate cancer screening[J]. J Health Commun. 2012, 17(6): 677-697.
[315] Dobransky K, Hargittai E. Inquiring minds acquiring wellness: uses of online and offline sources for health information[J]. Health Commun. 2012, 27(4): 331-343.

[316] Thomson M D, Siminoff L A, Longo D R. Internet use for prediagnosis symptom appraisal by colorectal cancer patients[J]. Health Educ Behav. 2012, 39(5): 583-588.
[317] Hohman K H, Price S N, Sonneville K, et al. Can the Internet be used to reach parents for family-based childhood obesity interventions?[J]. Clin Pediatr (Phila). 2012, 51(4): 314-320.
[318] Maddock C, Camporesi S, Lewis I, et al. Online information as a decision making aid for cancer patients: recommendations from the Eurocancercoms project[J]. Eur J Cancer. 2012, 48(7): 1055-1059.
[319] Han J Y, Kim J H, Yoon H J, et al. Social and psychological determinants of levels of engagement with an online breast cancer support group: posters, lurkers, and nonusers[J]. J Health Commun. 2012, 17(3): 356-371.
[320] Muse K, Mcmanus F, Leung C, et al. Cyberchondriasis: fact or fiction? A preliminary examination of the relationship between health anxiety and searching for health information on the Internet[J]. J Anxiety Disord. 2012, 26(1): 189-196.
[321] Bond M C, Klemt R, Merlis J, et al. Computer access and Internet use by urban and suburban emergency department customers[J]. J Emerg Med. 2012, 43(1): 159-165.
[322] Miller L M, Bell R A. Online health information seeking: the influence of age, information trustworthiness, and search challenges[J]. J Aging Health. 2012, 24(3): 525-541.
[323] Roncancio A M, Berenson A B, Rahman M. Health locus of control, acculturation, and health-related Internet use among Latinas[J]. J Health Commun. 2012, 17(6): 631-640.
[324] Alghamdi K M, Moussa N A. Internet use by the public to search for health-related information[J]. Int J Med Inform. 2012, 81(6): 363-373.
[325] Gee P M, Greenwood D A, Kim K K, et al. Exploration of the e-patient phenomenon in nursing informatics[J]. Nurs Outlook. 2012, 60(4): e9-e16.
[326] Leune A S, Nizard J.[Doctor Google: use of Internet during pregnancy in France in 2009][J]. J Gynecol Obstet Biol Reprod (Paris). 2012, 41(3): 243-254.
[327] Ossebaard H C, Seydel E R, van Gemert-Pijnen L. Online usability and patients with long-term conditions: a mixed-methods approach[J]. Int J Med Inform. 2012, 81(6): 374-387.
[328] Bui E, Rodgers R F, Herbert C, et al. The impact of internet coverage of the March 2011 Japan earthquake on sleep and posttraumatic stress symptoms: an international perspective[J]. Am J Psychiatry. 2012, 169(2): 221-222.
[329] Lee C J, Ramirez A S, Lewis N, et al. Looking beyond the Internet: examining socioeconomic inequalities in cancer information seeking among cancer patients[J]. Health Commun. 2012, 27(8): 806-817.
[330] Awad M A, Khalil I. Prediction of User's Web-Browsing Behavior: Application of Markov Model[J]. IEEE Trans Syst Man Cybern B Cybern. 2012.
[331] Valero-Aguilera B, Bermudez-Tamayo C, Garcia-Gutierrez J F, et al. Factors related to use of the Internet as a source of health information by urological cancer patients[J]. Support Care Cancer. 2012, 20(12): 3087-3094.
[332] Bernard E, Arnould M, Saint-Lary O, et al. Internet use for information seeking in clinical practice: a cross-sectional survey among French general practitioners[J]. Int J Med Inform. 2012, 81(7): 493-499.
[333] Ruiz-Canela M, Lopez-Del B C, Carlos S, et al.[Family, friends, and other sources of information associated with the initiation of sexual relations by adolescents in El Salvador][J].

Rev Panam Salud Publica. 2012, 31(1): 54-61.
[334] Kowalczyk N, Draper L J. Trends in patient information preferences and acquisition[J]. Radiol Technol. 2012, 83(4): 316-324.
[335] Kasabwala K, Agarwal N, Hansberry D R, et al. Readability assessment of patient education materials from the American Academy of Otolaryngology--Head and Neck Surgery Foundation[J]. Otolaryngol Head Neck Surg. 2012, 147(3): 466-471.
[336] Hogue M C, Doran E, Henry D A. A prompt to the web: the media and health information seeking behaviour[J]. PLoS One. 2012, 7(4): e34314.
[337] Horgan A, Sweeney J. University students' online habits and their use of the Internet for health information[J]. Comput Inform Nurs. 2012, 30(8): 402-408.
[338] Ettel G R, Nathanson I, Ettel D, et al. How do adolescents access health information? And do they ask their physicians?[J]. Perm J. 2012, 16(1): 35-38.
[339] Brockes C, Schenkel J S, Buehler R N, et al. Medical online consultation service regarding maxillofacial surgery[J]. J Craniomaxillofac Surg. 2012, 40(7): 626-630.
[340] Hu X, Bell R A, Kravitz R L, et al. The prepared patient: information seeking of online support group members before their medical appointments[J]. J Health Commun. 2012, 17(8): 960-978.
[341] Davis N F, Smyth L G, Flood H D. Detecting internet activity for erectile dysfunction using search engine query data in the Republic of Ireland[J]. BJU Int. 2012, 110(11 Pt C): E939-E942.
[342] Mesch G, Mano R, Tsamir J. Minority status and health information search: a test of the social diversification hypothesis[J]. Soc Sci Med. 2012, 75(5): 854-858.
[343] Schnack D.[Empowered patients? "Dubious information" from the web complicate physician-patient dialogue][J]. MMW Fortschr Med. 2012, 154(6): 14.
[344] Parks A C, Della P M, Pierce R S, et al. Pursuing happiness in everyday life: the characteristics and behaviors of online happiness seekers[J]. Emotion. 2012, 12(6): 1222-1234.
[345] Geana M V, Daley C M, Nazir N, et al. Use of online health information resources by American Indians and Alaska Natives[J]. J Health Commun. 2012, 17(7): 820-835.
[346] Kirthi V, Modi B N. Coronary angioplasty and the internet: what can patients searching online expect to find?[J]. J Interv Cardiol. 2012, 25(5): 476-481.
[347] Feufel M A, Stahl S F. What do web-use skill differences imply for online health information searches?[J]. J Med Internet Res. 2012, 14(3): e87.
[348] Talarczyk J, Hauke J, Poniewaz M, et al.[Internet as a source of information about infertility among infertile patients][J]. Ginekol Pol. 2012, 83(4): 250-254.
[349] D'Agostino T A, Ostroff J S, Heerdt A, et al. Toward a greater understanding of breast cancer patients' decisions to discuss cancer-related internet information with their doctors: an exploratory study[J]. Patient Educ Couns. 2012, 89(1): 109-115.
[350] Holtgrafe C, Zentes J. Multifaceted determinants of online non-prescription drug information seeking and the impact on consumers' use of purchase channels[J]. Health Informatics J. 2012, 18(2): 95-110.
[351] Chen A T. Information seeking over the course of illness: the experience of people with fibromyalgia[J]. Musculoskeletal Care. 2012, 10(4): 212-220.
[352] Gulliver A, Griffiths K M, Christensen H, et al. Internet-based interventions to promote

mental health help-seeking in elite athletes: an exploratory randomized controlled trial[J]. J Med Internet Res. 2012, 14(3): e69.
[353] Moreno M A, Whitehill J M. New media, old risks: toward an understanding of the relationships between online and offline health behavior[J]. Arch Pediatr Adolesc Med. 2012, 166(9): 868-869.
[354] Gross M, Goode V H. The informationist's role in 21st century medicine[J]. Md Med. 2012, 13(1): 27-28.
[355] Smith C E, Massey-Stokes M, Lieberth A. Health information needs of d/Deaf adolescent females: a call to action[J]. Am Ann Deaf. 2012, 157(1): 41-47.
[356] Diamantidis C J, Zuckerman M, Fink W, et al. Usability of a CKD educational website targeted to patients and their family members[J]. Clin J Am Soc Nephrol. 2012, 7(10): 1553-1560.
[357] Sueki H. Association between deliberate self-harm-related Internet searches and the mental states and lifetime suicidal behaviors of Japanese young adults[J]. Psychiatry Clin Neurosci. 2012, 66(5): 451-453.
[358] Korb-Savoldelli V, Gillaizeau F, Caruba T, et al. Information about medication in HIV-infected patients and its relation to adherence[J]. Swiss Med Wkly. 2012, 142: w13642.
[359] Zajac I T, Flight I H, Wilson C, et al. Internet usage and openness to internet-delivered health information among Australian adults aged over 50 years[J]. Australas Med J. 2012, 5(5): 262-267.
[360] Finney R L, Hesse B W, Moser R P, et al. Socioeconomic and geographic disparities in health information seeking and Internet use in Puerto Rico[J]. J Med Internet Res. 2012, 14(4): e104.
[361] Lewis S P, Rosenrot S A, Messner M A. Seeking validation in unlikely places: the nature of online questions about non-suicidal self-injury[J]. Arch Suicide Res. 2012, 16(3): 263-272.
[362] Pletneva N, Vargas A, Kalogianni K, et al. Online health information search: what struggles and empowers the users? Results of an online survey[J]. Stud Health Technol Inform. 2012, 180: 843-847.
[363] Eklund A M. Tracking changes in search behaviour at a health web site[J]. Stud Health Technol Inform. 2012, 180: 858-862.
[364] Wang H, Masuda T, Ito K, et al. How much information? East Asian and North American cultural products and information search performance[J]. Pers Soc Psychol Bull. 2012, 38(12): 1539-1551.
[365] Kogan L R, Schoenfeld-Tacher R, Viera A R. The Internet and health information: differences in pet owners based on age, gender, and education[J]. J Med Libr Assoc. 2012, 100(3): 197-204.
[366] Sakai Y, Kunimoto C, Kurata K. Health information seekers in Japan: a snapshot of needs, behavior, and recognition in 2008[J]. J Med Libr Assoc. 2012, 100(3): 205-213.
[367] Suziedelyte A. How does searching for health information on the Internet affect individuals' demand for health care services?[J]. Soc Sci Med. 2012, 75(10): 1828-1835.
[368] Wong S, Walker J R, Carr R, et al. The information needs and preferences of persons with longstanding inflammatory bowel disease[J]. Can J Gastroenterol. 2012, 26(8): 525-531.
[369] Henshaw H, Clark D P, Kang S, et al. Computer skills and internet use in adults aged

50-74 years: influence of hearing difficulties[J]. J Med Internet Res. 2012, 14(4): e113.
[370] Kavlak O, Atan S U, Gulec D, et al. Pregnant women's use of the internet in relation to their pregnancy in Izmir, Turkey[J]. Inform Health Soc Care. 2012, 37(4): 253-263.
[371] Hardoff D, Friedman R, Pilo N, et al.[Adolescents ask physicians on the Internet: a one-year survey of adolescents' questions on health issues in an Internet forum][J]. Harefuah. 2012, 151(6): 338-341, 379.
[372] Ofran Y, Paltiel O, Pelleg D, et al. Patterns of information-seeking for cancer on the internet: an analysis of real world data[J]. PLoS One. 2012, 7(9): e45921.
[373] Donelle L, Booth R G. Health tweets: an exploration of health promotion on twitter[J]. Online J Issues Nurs. 2012, 17(3): 4.
[374] Magee R G, Wojdynski B W. Mortality salience effects on selective exposure and web browsing behavior[J]. Cyberpsychol Behav Soc Netw. 2012, 15(12): 663-668.
[375] Littleford J, Gottschalk T. The medical student's Toolkit[J]. Med Educ. 2012, 46(11): 1117.
[376] Borracci R A, Manente D, Giorgi M A, et al.[Patients' preferences for information in health care decision-making][J]. Medicina (B Aires). 2012, 72(5): 393-398.
[377] Charbonneau D H. Readability of menopause web sites: a cross-sectional study[J]. J Women Aging. 2012, 24(4): 280-291.
[378] Kim A S, Poisson S N, Easton J D, et al. A cross-sectional study of individuals seeking information on transient ischemic attack and stroke symptoms online: a target for intervention?[J]. PLoS One. 2012, 7(10): e47997.
[379] Duplaga M. Acceptance of Internet-based health care services among households in Poland: secondary analysis of a population-based survey[J]. J Med Internet Res. 2012, 14(6): e164.
[380] Jansen L, Rasekaba T, Presnell S, et al. Finding evidence to support practice in allied health: peers, experience, and the internet[J]. J Allied Health. 2012, 41(4): 154-161.
[381] Marino R, Habibi E, Morgan M, et al. Information and communication technology use among Victorian and South Australian oral health professions students[J]. J Dent Educ. 2012, 76(12): 1667-1674.
[382] Zheluk A, Gillespie J A, Quinn C. Searching for truth: internet search patterns as a method of investigating online responses to a Russian illicit drug policy debate[J]. J Med Internet Res. 2012, 14(6): e165.
[383] van Velsen L, van Gemert-Pijnen J E, Beaujean D J, et al. Should health organizations use web 2.0 media in times of an infectious disease crisis? An in-depth qualitative study of citizens' information behavior during an EHEC outbreak[J]. J Med Internet Res. 2012, 14(6): e181.
[384] Chisholm R, Finnell J T. Emergency department physician internet use during clinical encounters[J]. AMIA Annu Symp Proc. 2012, 2012: 1176-1183.
[385] Dixon B E, Kaneshiro K. Improving Access to HIV and AIDS Information Resources for Patients, Caregivers, and Clinicians: Results from the SHINE Project[J]. Online J Public Health Inform. 2012, 4(1).
[386] Paquin R S, Richards A S, Koehly L M, et al. Exploring dispositional tendencies to seek online information about direct-to-consumer genetic testing[J]. Transl Behav Med. 2012, 2(4): 392-400.

[387] Willard S D, Nguyen M M. Internet search trends analysis tools can provide real-time data on kidney stone disease in the United States[J]. Urology. 2013, 81(1): 37-42.
[388] Emond Y, de Groot J, Wetzels W, et al. Internet guidance in oncology practice: determinants of health professionals' Internet referral behavior[J]. Psychooncology. 2013, 22(1): 74-82.
[389] White R, Horvitz E. From web search to healthcare utilization: privacy-sensitive studies from mobile data[J]. J Am Med Inform Assoc. 2013, 20(1): 61-68.
[390] Kratzke C, Wilson S, Vilchis H. Reaching rural women: breast cancer prevention information seeking behaviors and interest in Internet, cell phone, and text use[J]. J Community Health. 2013, 38(1): 54-61.
[391] Walker K K. Bytes, and pixels and pieces of information[J]. Health Commun. 2013, 28(3): 314-316.
[392] Lee J Y, Sundar S S. To tweet or to retweet? That is the question for health professionals on twitter[J]. Health Commun. 2013, 28(5): 509-524.
[393] Olsen L L, Kruse S, Brussoni M. Unheard voices: a qualitative exploration of fathers' access of child safety information[J]. J Community Health. 2013, 38(1): 187-194.
[394] Mukewar S, Mani P, Wu X, et al. YouTube and inflammatory bowel disease[J]. J Crohns Colitis. 2013, 7(5): 392-402.
[395] Abt S A, Pablo H S, Serrano A P, et al.[Information needs and internet use in patients with breast cancer in Spain][J]. Gac Sanit. 2013, 27(3): 241-247.
[396] Gao L L, Larsson M, Luo S Y. Internet use by Chinese women seeking pregnancy-related information[J]. Midwifery. 2013, 29(7): 730-735.
[397] Markey P M, Markey C N. Annual variation in Internet keyword searches: Linking dieting interest to obesity and negative health outcomes[J]. J Health Psychol. 2013, 18(7): 875-886.
[398] Selsky C, Luta G, Noone A M, et al. Internet access and online cancer information seeking among Latino immigrants from safety net clinics[J]. J Health Commun. 2013, 18(1): 58-70.
[399] Huberty J, Dinkel D, Beets M W, et al. Describing the use of the internet for health, physical activity, and nutrition information in pregnant women[J]. Matern Child Health J. 2013, 17(8): 1363-1372.
[400] Naftel R P, Safiano N A, Falola M I, et al. Technology preferences among caregivers of children with hydrocephalus[J]. J Neurosurg Pediatr. 2013, 11(1): 26-36.
[401] Matura L A, Mcdonough A, Aglietti L M, et al. A virtual community: concerns of patients with pulmonary hypertension[J]. Clin Nurs Res. 2013, 22(2): 155-171.
[402] Bosley J C, Zhao N W, Hill S, et al. Decoding twitter: Surveillance and trends for cardiac arrest and resuscitation communication[J]. Resuscitation. 2013, 84(2): 206-212.
[403] Russo J A, Parisi S M, Kukla K, et al. Women's information-seeking behavior after receiving contraceptive versus noncontraceptive prescriptions[J]. Contraception. 2013, 87(6): 824-829.
[404] Henderson E M, Keogh E, Rosser B A, et al. Searching the internet for help with pain: adolescent search, coping, and medication behaviour[J]. Br J Health Psychol. 2013, 18(1): 218-232.
[405] Laz T H, Berenson A B. Racial and ethnic disparities in internet use for seeking health

information among young women[J]. J Health Commun. 2013, 18(2): 250-260.
[406] Linnman C, Maleki N, Becerra L, et al. Migraine tweets - what can online behavior tell us about disease?[J]. Cephalalgia. 2013, 33(1): 68-69.
[407] Sadasivam R S, Kinney R L, Lemon S C, et al. Internet health information seeking is a team sport: analysis of the Pew Internet Survey[J]. Int J Med Inform. 2013, 82(3): 193-200.
[408] Filippi M K, Mccloskey C, Williams C, et al. Perceptions, barriers, and suggestions for creation of a tobacco and health website among American Indian/Alaska Native college students[J]. J Community Health. 2013, 38(3): 486-491.
[409] Gunn J R, Lester D. Using google searches on the internet to monitor suicidal behavior[J]. J Affect Disord. 2013, 148(2-3): 411-412.
[410] Ranney M L, Daya M. Twitter and resuscitation education: is this the future?[J]. Resuscitation. 2013, 84(2): 147-148.
[411] Gosselin M M, Mulcahey M K, Feller E, et al. Examining Internet resources on gender differences in ACL injuries: what patients are reading[J]. Knee. 2013, 20(3): 196-202.
[412] De Leo J A, Wulfert E. Problematic Internet use and other risky behaviors in college students: an application of problem-behavior theory[J]. Psychol Addict Behav. 2013, 27(1): 133-141.
[413] Kayhan V O. Seeking health information on the web: positive hypothesis testing[J]. Int J Med Inform. 2013, 82(4): 268-275.
[414] Seymour-Smith S. A reconsideration of the gendered mechanisms of support in online interactions about testicular implants: a discursive approach[J]. Health Psychol. 2013, 32(1): 91-99.
[415] Yli-Uotila T, Rantanen A, Suominen T. Motives of cancer patients for using the Internet to seek social support[J]. Eur J Cancer Care (Engl). 2013, 22(2): 261-271.
[416] Abt S A.[PyDEsalud platform: patients' experience, information, support and health capacitation][J]. Med Clin (Barc). 2013, 140(12): 544-545.
[417] Harland N, Drew B T. A survey investigation of UK physiotherapists' use of online search engines for continuing professional development[J]. Physiotherapy. 2013, 99(3): 201-206.
[418] Peters S L, Lind J N, Humphrey J R, et al. Safe lists for medications in pregnancy: inadequate evidence base and inconsistent guidance from Web-based information, 2011[J]. Pharmacoepidemiol Drug Saf. 2013, 22(3): 324-328.
[419] Thackeray R, Crookston B T, West J H. Correlates of health-related social media use among adults[J]. J Med Internet Res. 2013, 15(1): e21.
[420] Starcevic V, Berle D. Cyberchondria: towards a better understanding of excessive health-related Internet use[J]. Expert Rev Neurother. 2013, 13(2): 205-213.
[421] Colon Y. Searching for pain information, education, and support on the Internet[J]. J Pain Palliat Care Pharmacother. 2013, 27(1): 71-73.
[422] Buck H G. Can you find reliable information about EOL on the Internet?[J]. Nursing. 2013, 43(3): 16-17.
[423] Mortensen H J, Alexander J L, Nehrenz G M, et al. Infection control professionals' information-seeking preferences[J]. Health Info Libr J. 2013, 30(1): 23-34.
[424] Johnson C. Survey finds physicians very wary of doctor ratings[J]. Physician Exec. 2013, 39(1): 6-8, 10, 12.

[425] Kuehn B M. More than one-third of US individuals use the Internet to self-diagnose[J]. JAMA. 2013, 309(8): 756-757.
[426] Manafo E, Wong S. Promoting eHealth literacy in older adults: key informant perspectives[J]. Can J Diet Pract Res. 2013, 74(1): 37-41.
[427] Blumenstein I, Mcdermott E, Keegan D, et al. Sources of information and factual knowledge in Europeans with inflammatory bowel diseases: a cross-cultural comparison between German and Irish patients[J]. J Crohns Colitis. 2013, 7(9): e331-e336.
[428] Oprescu F, Campo S, Lowe J, et al. Online information exchanges for parents of children with a rare health condition: key findings from an online support community[J]. J Med Internet Res. 2013, 15(1): e16.
[429] Klemenc-Ketis Z, Kersnik J. Seeking health advice on the Internet in patients with health problems: a cross-sectional population study in Slovenia[J]. Inform Health Soc Care. 2013, 38(3): 280-290.
[430] Maloni J A, Przeworski A, Damato E G. Web recruitment and internet use and preferences reported by women with postpartum depression after pregnancy complications[J]. Arch Psychiatr Nurs. 2013, 27(2): 90-95.
[431] Chung J E. Patient-provider discussion of online health information: results from the 2007 Health Information National Trends Survey (HINTS)[J]. J Health Commun. 2013, 18(6): 627-648.
[432] Ayers J W, Althouse B M, Allem J P, et al. Seasonality in seeking mental health information on Google[J]. Am J Prev Med. 2013, 44(5): 520-525.
[433] Connor K, Brady R R, Tulloh B, et al. Smartphone applications (apps) for bariatric surgery[J]. Obes Surg. 2013, 23(10): 1669-1672.
[434] Rozensky R H, Tovian S M, Gartley C B, et al. A quality of life survey of individuals with urinary incontinence who visit a self-help website: implications for those seeking healthcare information[J]. J Clin Psychol Med Settings. 2013, 20(3): 275-283.
[435] Hand F, Mcdowell D T, Glynn R W, et al. Patterns of internet use by parents of children attending a pediatric surgical service[J]. Pediatr Surg Int. 2013, 29(7): 729-733.
[436] Nordfeldt S, Angarne-Lindberg T, Nordwall M, et al. Parents of adolescents with type 1 diabetes--their views on information and communication needs and internet use. A qualitative study[J]. PLoS One. 2013, 8(4): e62096.
[437] Braun T, Harreus U. Medical nowcasting using Google Trends: application in otolaryngology[J]. Eur Arch Otorhinolaryngol. 2013, 270(7): 2157-2160.
[438] Choi N G, Dinitto D M. The digital divide among low-income homebound older adults: Internet use patterns, eHealth literacy, and attitudes toward computer/Internet use[J]. J Med Internet Res. 2013, 15(5): e93.
[439] Li Y, Polk J, Plankey M. Online health-searching behavior among HIV-seropositive and HIV-seronegative men who have sex with men in the Baltimore and Washington, DC area[J]. J Med Internet Res. 2013, 15(5): e78.
[440] Vandelanotte C, Caperchione C M, Ellison M, et al. What kinds of website and mobile phone-delivered physical activity and nutrition interventions do middle-aged men want?[J]. J Health Commun. 2013, 18(9): 1070-1083.
[441] Lau A Y, Proudfoot J, Andrews A, et al. Which bundles of features in a Web-based personally controlled health management system are associated with consumer help-seeking

behaviors for physical and emotional well-being?[J]. J Med Internet Res. 2013, 15(5): e79.
[442] Kuehn B M. Scientists mine web search data to identify epidemics and adverse events[J]. JAMA. 2013, 309(18): 1883-1884.
[443] Thoren E S, Oberg M, Wanstrom G, et al. Internet access and use in adults with hearing loss[J]. J Med Internet Res. 2013, 15(5): e91.
[444] Sheng X, Simpson P M. Seniors, health information, and the Internet: motivation, ability, and Internet knowledge[J]. Cyberpsychol Behav Soc Netw. 2013, 16(10): 740-746.
[445] Choi N G, Dinitto D M. Internet use among older adults: association with health needs, psychological capital, and social capital[J]. J Med Internet Res. 2013, 15(5): e97.
[446] Burton-Jeangros C, Hammer R.[Information seeking on the internet: what information are pregnant women seeking?][J]. Rev Med Suisse. 2013, 9(383): 895-897.
[447] Lo S C. Evaluating ecommerce websites cognitive efficiency: an integrative framework based on data envelopment analysis[J]. Appl Ergon. 2013, 44(6): 1004-1014.
[448] van den Bree M B, Miller G, Mansell E, et al. The internet is parents' main source of information about psychiatric manifestations of 22q11.2 deletion syndrome (22q11.2DS)[J]. Eur J Med Genet. 2013, 56(8): 439-441.
[449] Abbas A D, Abubakar A M, Omeiza B, et al. Information-seeking behavior and computer literacy among resident doctors in Maiduguri, Nigeria[J]. Ann Afr Med. 2013, 12(2): 115-119.
[450] Dunstone K, Makin J, Conway C. Monitoring public interest in solariums: variations in internet search volumes over time in Victoria[J]. Aust N Z J Public Health. 2013, 37(3): 292.
[451] Richardson C G, Hamadani L G, Gotay C. Quantifying Canadians' use of the Internet as a source of information on behavioural risk factor modifications related to cancer prevention[J]. Chronic Dis Inj Can. 2013, 33(3): 123-128.
[452] Hechanova M R, Tuliao A P, Teh L A, et al. Problem severity, technology adoption, and intent to seek online counseling among overseas Filipino workers[J]. Cyberpsychol Behav Soc Netw. 2013, 16(8): 613-617.
[453] Timimi F K. The shape of digital engagement: health care and social media[J]. J Ambul Care Manage. 2013, 36(3): 187-192.
[454][Google and co. replace grandparents. 90 percent of children search the web for answers][J]. Kinderkrankenschwester. 2013, 32(5): 192.
[455] Marya C M, Marya K M, Dahiya V, et al. Internet usage among dental students in north India[J]. J Pak Med Assoc. 2013, 63(5): 628-629.
[456] Thoren E M, Metze B, Buhrer C, et al. Online support for parents of preterm infants: a qualitative and content analysis of Facebook 'preemie' groups[J]. Arch Dis Child Fetal Neonatal Ed. 2013, 98(6): F534-F538.
[457] De Freitas J, Falls B A, Haque O S, et al. Vulnerabilities to misinformation in online pharmaceutical marketing[J]. J R Soc Med. 2013, 106(5): 184-189.
[458] Sweet S N, Perrier M J, Podzyhun C, et al. Identifying physical activity information needs and preferred methods of delivery of people with multiple sclerosis[J]. Disabil Rehabil. 2013, 35(24): 2056-2063.
[459] Zhao J, Ha S, Widdows R. Building trusting relationships in online health communities[J]. Cyberpsychol Behav Soc Netw. 2013, 16(9): 650-657.
[460] Mestrovic T, Ozegic O, Bujas I. EEG cerebral dysrhythmia in non-epileptic individuals

as an incentive for seeking online health consultation[J]. J Postgrad Med. 2013, 59(2): 163-164.
[461] Kritz M, Gschwandtner M, Stefanov V, et al. Utilization and perceived problems of online medical resources and search tools among different groups of European physicians[J]. J Med Internet Res. 2013, 15(6): e122.
[462] Nordfeldt S, Angarne-Lindberg T, Nordwall M, et al. As Facts and Chats Go Online, What Is Important for Adolescents with Type 1 Diabetes?[J]. PLoS One. 2013, 8(6): e67659.
[463] Levesque L. Online information trap[J]. Nursing. 2013, 43(6): 8.
[464] Neumark Y, Lopez-Quintero C, Feldman B S, et al. Online health information seeking among Jewish and Arab adolescents in Israel: results from a national school survey[J]. J Health Commun. 2013, 18(9): 1097-1115.
[465] Huesch M D. Privacy threats when seeking online health information[J]. JAMA Intern Med. 2013, 173(19): 1838-1839.
[466] Drushel B E. HIV/AIDS, social capital, and online social networks[J]. J Homosex. 2013, 60(8): 1230-1249.
[467] Arai T, Fuji K, Yoshida F.[Determinants of information-seeking about crime and crime prevention: information-seeking on the Internet][J]. Shinrigaku Kenkyu. 2013, 84(2): 83-92.
[468] Kostkova P, Fowler D, Wiseman S, et al. Major infection events over 5 years: how is media coverage influencing online information needs of health care professionals and the public?[J]. J Med Internet Res. 2013, 15(7): e107.
[469] Goldner M, Hale T M, Cotten S R, et al. The intersection of gender and place in online health activities[J]. J Health Commun. 2013, 18(10): 1235-1255.
[470] Mccully S N, Don B P, Updegraff J A. Using the Internet to help with diet, weight, and physical activity: results from the Health Information National Trends Survey (HINTS)[J]. J Med Internet Res. 2013, 15(8): e148.
[471] Rai A, Chen L, Pye J, et al. Understanding determinants of consumer mobile health usage intentions, assimilation, and channel preferences[J]. J Med Internet Res. 2013, 15(8): e149.
[472] Gabbert T I, Metze B, Buhrer C, et al. Use of social networking sites by parents of very low birth weight infants: experiences and the potential of a dedicated site[J]. Eur J Pediatr. 2013, 172(12): 1671-1677.
[473] Medlock S, Eslami S, Askari M, et al. The consequences of seniors seeking health information using the internet and other sources[J]. Stud Health Technol Inform. 2013, 192: 457-460.
[474] Samwald M, Kritz M, Gschwandtner M, et al. Physicians searching the web for medical question answering: a European survey and local user studies[J]. Stud Health Technol Inform. 2013, 192: 1103.
[475] Rollin L, Ladner J, Gislard A, et al. Hazard information needs and information seeking in French workers[J]. Occup Med (Lond). 2013, 63(7): 473-478.
[476] Chaudhuri S, Le T, White C, et al. Examining health information-seeking behaviors of older adults[J]. Comput Inform Nurs. 2013, 31(11): 547-553.
[477] Justumus P, Colby D, Mai D A T, et al. Willingness to use the Internet to seek information on HIV prevention and care among men who have sex with men in Ho Chi Minh City, Vietnam[J]. PLoS One. 2013, 8(8): e71471.

[478] Fergus T A. Cyberchondria and intolerance of uncertainty: examining when individuals experience health anxiety in response to Internet searches for medical information[J]. Cyberpsychol Behav Soc Netw. 2013, 16(10): 735-739.
[479] Sacks S, Abenhaim H A. How evidence-based is the information on the internet about nausea and vomiting of pregnancy?[J]. J Obstet Gynaecol Can. 2013, 35(8): 697-703.
[480] Mishra M V, Bennett M, Vincent A, et al. Identifying barriers to patient acceptance of active surveillance: content analysis of online patient communications[J]. PLoS One. 2013, 8(9): e68563.
[481] Tong S T, Heinemann-Lafave D, Jeon J, et al. The use of pro-ana blogs for online social support[J]. Eat Disord. 2013, 21(5): 408-422.
[482] Bianco A, Zucco R, Nobile C G, et al. Parents seeking health-related information on the Internet: cross-sectional study[J]. J Med Internet Res. 2013, 15(9): e204.
[483] Bert F, Gualano M R, Brusaferro S, et al. Pregnancy e-health: a multicenter Italian cross-sectional study on Internet use and decision-making among pregnant women[J]. J Epidemiol Community Health. 2013, 67(12): 1013-1018.
[484] Tielemans M M, van Oijen M G. Online follow-up of individuals with gastroesophageal reflux disease using a patient-reported outcomes instrument: results of an observational study[J]. BMC Gastroenterol. 2013, 13: 144.
[485] Van de Belt T H, Engelen L J, Berben S A, et al. Internet and social media for health-related information and communication in health care: preferences of the Dutch general population[J]. J Med Internet Res. 2013, 15(10): e220.
[486] Hardoff D. Health issues in adolescents' Internet use - benefits and risks[J]. Georgian Med News. 2013(222): 99-103.
[487] Miller C J, Neuhaus I M, Sobanko J F, et al. Accuracy and completeness of patient information in organic World-Wide Web search for Mohs surgery: a prospective cross-sectional multirater study using consensus criteria[J]. Dermatol Surg. 2013, 39(11): 1654-1661.
[488] Dudas R A, Crocetti M. Pediatric caregiver attitudes toward email communication: survey in an urban primary care setting[J]. J Med Internet Res. 2013, 15(10): e228.
[489] Otte W M, van Diessen E, Bell G S, et al. Web-search trends shed light on the nature of lunacy: relationship between moon phases and epilepsy information-seeking behavior[J]. Epilepsy Behav. 2013, 29(3): 571-573.
[490] Calvert J K, Aidala A A, West J H. An Ecological View of Internet Health Information Seeking Behavior Predictors: Findings from the CHAIN Study[J]. Open AIDS J. 2013, 7: 42-46.
[491] Gilbert M, Hottes T S, Kerr T, et al. Factors associated with intention to use internet-based testing for sexually transmitted infections among men who have sex with men[J]. J Med Internet Res. 2013, 15(11): e254.
[492] Kearney T E, Lieu D, Singer N, et al. Investigating the reliability of substance toxicity information found on the Internet in pediatric poisonings[J]. Pediatr Emerg Care. 2013, 29(12): 1249-1254.
[493] Zilincik M, Navrat P, Koskova G. Exploratory search on Twitter utilizing user feedback and multi-perspective microblog analysis[J]. PLoS One. 2013, 8(11): e78857.
[494] Zhang Y. The effects of preference for information on consumers' online health

information search behavior[J]. J Med Internet Res. 2013, 15(11): e234.
[495] Sobkowicz P. Quantitative agent based model of user behavior in an Internet discussion forum[J]. PLoS One. 2013, 8(12): e80524.
[496] Shaffer V A, Owens J, Zikmund-Fisher B J. The effect of patient narratives on information search in a web-based breast cancer decision aid: an eye-tracking study[J]. J Med Internet Res. 2013, 15(12): e273.
[497] Slauson-Blevins K S, Mcquillan J, Greil A L. Online and in-person health-seeking for infertility[J]. Soc Sci Med. 2013, 99: 110-115.
[498] Kim S C, Shah D V, Namkoong K, et al. Predictors of Online Health Information Seeking Among Women with Breast Cancer: The Role of Social Support Perception and Emotional Well-Being[J]. J Comput Mediat Commun. 2013, 18(2): 98-118.
[499] Jones R. Development of a Questionnaire and Cross-Sectional Survey of Patient eHealth Readiness and eHealth Inequalities[J]. Med 2 0. 2013, 2(2): e9.
[500] Yang Y T, Horneffer M, Dilisio N. Mining social media and web searches for disease detection[J]. J Public Health Res. 2013, 2(1): 17-21.
[501] Chung J E. Social networking in online support groups for health: how online social networking benefits patients[J]. J Health Commun. 2014, 19(6): 639-659.
[502] White R W, Horvitz E. From health search to healthcare: explorations of intention and utilization via query logs and user surveys[J]. J Am Med Inform Assoc. 2014, 21(1): 49-55.
[503] Gutierrez N, Kindratt T B, Pagels P, et al. Health literacy, health information seeking behaviors and internet use among patients attending a private and public clinic in the same geographic area[J]. J Community Health. 2014, 39(1): 83-89.
[504] Penta M A, Baban A. Dangerous agent or saviour? HPV vaccine representations on online discussion forums in Romania[J]. Int J Behav Med. 2014, 21(1): 20-28.
[505] Huh J, Shin W. Trust in prescription drug brand websites: website trust cues, attitude toward the website, and behavioral intentions[J]. J Health Commun. 2014, 19(2): 170-191.
[506] Park H, Park M S. Cancer information-seeking behaviors and information needs among Korean Americans in the online community[J]. J Community Health. 2014, 39(2): 213-220.
[507] Cunningham C E, Walker J R, Eastwood J D, et al. Modeling mental health information preferences during the early adult years: a discrete choice conjoint experiment[J]. J Health Commun. 2014, 19(4): 413-440.
[508] Harsha A K, Schmitt J E, Stavropoulos S W. Know your market: use of online query tools to quantify trends in patient information-seeking behavior for varicose vein treatment[J]. J Vasc Interv Radiol. 2014, 25(1): 53-57.
[509] Holloway I W, Rice E, Gibbs J, et al. Acceptability of smartphone application-based HIV prevention among young men who have sex with men[J]. AIDS Behav. 2014, 18(2): 285-296.
[510] Chang S J, Im E O. A path analysis of Internet health information seeking behaviors among older adults[J]. Geriatr Nurs. 2014, 35(2): 137-141.
[511] Costello K L, Murillo A P. "I want your kidney!" Information seeking, sharing, and disclosure when soliciting a kidney donor online[J]. Patient Educ Couns. 2014, 94(3): 423-426.
[512] Brigo F, Igwe S C, Ausserer H, et al. Why do people Google epilepsy? An infodemiological study of online behavior for epilepsy-related search terms[J]. Epilepsy

Behav. 2014, 31: 67-70.
[513] Nahai F. Anonymous sources[J]. Aesthet Surg J. 2014, 34(1): 189-191.
[514] Boruff J T, Storie D. Mobile devices in medicine: a survey of how medical students, residents, and faculty use smartphones and other mobile devices to find information[J]. J Med Libr Assoc. 2014, 102(1): 22-30.
[515] Kogan L R, Schoenfeld-Tacher R, Gould L, et al. Providing an information prescription in veterinary medical clinics: a pilot study[J]. J Med Libr Assoc. 2014, 102(1): 41-46.
[516] Mitchell S J, Godoy L, Shabazz K, et al. Internet and mobile technology use among urban African American parents: survey study of a clinical population[J]. J Med Internet Res. 2014, 16(1): e9.
[517] Luger T M, Houston T K, Suls J. Older adult experience of online diagnosis: results from a scenario-based think-aloud protocol[J]. J Med Internet Res. 2014, 16(1): e16.
[518] Briet J P, Hageman M G, Blok R, et al. When do patients with hand illness seek online health consultations and what do they ask?[J]. Clin Orthop Relat Res. 2014, 472(4): 1246-1250.
[519] Magnezi R, Grosberg D, Novikov I, et al. Characteristics of patients seeking health information online via social health networks versus general Internet sites: a comparative study[J]. Inform Health Soc Care. 2014.
[520] Schook R M, Linssen C, Schramel F M, et al. Why do patients and caregivers seek answers from the Internet and online lung specialists? A qualitative study[J]. J Med Internet Res. 2014, 16(2): e37.
[521] Salgado M V, Mejia R, Kaplan C P, et al. Smoking behavior and use of tobacco industry sponsored websites among medical students and young physicians in Argentina[J]. J Med Internet Res. 2014, 16(2): e35.
[522] Kahlon M, Yuan L, Daigre J, et al. The use and significance of a research networking system[J]. J Med Internet Res. 2014, 16(2): e46.
[523] Kim J N, Lee S. Communication and cybercoping: coping with chronic illness through communicative action in online support networks[J]. J Health Commun. 2014, 19(7): 775-794.
[524] Yom-Tov E, White R W, Horvitz E. Seeking insights about cycling mood disorders via anonymized search logs[J]. J Med Internet Res. 2014, 16(2): e65.
[525] Kauer S D, Mangan C, Sanci L. Do online mental health services improve help-seeking for young people? A systematic review[J]. J Med Internet Res. 2014, 16(3): e66.
[526] O'Neill B, Ziebland S, Valderas J, et al. User-generated online health content: a survey of Internet users in the United Kingdom[J]. J Med Internet Res. 2014, 16(4): e118.
[527] Beck F, Richard J B, Nguyen-Thanh V, et al. Use of the internet as a health information resource among French young adults: results from a nationally representative survey[J]. J Med Internet Res. 2014, 16(5): e128.
[528] Kontos E, Blake K D, Chou W Y, et al. Predictors of eHealth usage: insights on the digital divide from the Health Information National Trends Survey 2012[J]. J Med Internet Res. 2014, 16(7): e172.
[529] Seckin G. Health information on the web and consumers' perspectives on health professionals' responses to information exchange[J]. Med 2 0. 2014, 3(2): e4.
[530] Lee Y J, Boden-Albala B, Larson E, et al. Online health information seeking behaviors

of Hispanics in New York City: a community-based cross-sectional study[J]. J Med Internet Res. 2014, 16(7): e176.
[531] Bindhim N F, Mcgeechan K, Trevena L. Who Uses Smoking Cessation Apps? A Feasibility Study Across Three Countries via Smartphones[J]. JMIR Mhealth Uhealth. 2014, 2(1): e4.
[532] Kowalski C, Kahana E, Kuhr K, et al. Changes over time in the utilization of disease-related internet information in newly diagnosed breast cancer patients 2007 to 2013[J]. J Med Internet Res. 2014, 16(8): e195.
[533] Lerouge C, Van Slyke C, Seale D, et al. Baby boomers' adoption of consumer health technologies: survey on readiness and barriers[J]. J Med Internet Res. 2014, 16(9): e200.
